# Senescence-associated morphological profiles (SAMPs): an image-based phenotypic profiling method for evaluating the inter and intra model heterogeneity of senescence

**DOI:** 10.18632/aging.204072

**Published:** 2022-05-16

**Authors:** Ryan Wallis, Deborah Milligan, Bethany Hughes, Hannah Mizen, José Alberto López-Domínguez, Ugochim Eduputa, Eleanor J. Tyler, Manuel Serrano, Cleo L. Bishop

**Affiliations:** 1 Blizard Institute of Cell and Molecular Science, Barts and The London School of Medicine and Dentistry, Queen Mary University of London, London, UK; 2 Institute for Research in Biomedicine (IRB Barcelona), Barcelona Institute of Science and Technology (BIST), Barcelona, Spain; 3 Catalan Institution for Research and Advanced Studies (ICREA), Barcelona, Spain

**Keywords:** senescence, high content profiling of senescence hallmarks, morphology, senescence-associated morphological profiles (SAMPs)

## Abstract

Senescence occurs in response to a number of damaging stimuli to limit oncogenic transformation and cancer development. As no single, universal senescence marker has been discovered, the confident classification of senescence induction requires the parallel assessment of a series of hallmarks. Therefore, there is a growing need for “first-pass” tools of senescence identification to streamline experimental workflows and complement conventional markers.

Here, we utilise a high content, multidimensional phenotypic profiling-based approach, to assess the morphological profiles of senescent cells induced via a range of stimuli. In the context of senescence, we refer to these as senescence-associated morphological profiles (SAMPs), as they facilitate distinction between senescent and proliferating cells. The complexity of the profiles generated also allows exploration of the heterogeneity both between models of senescence and within an individual senescence model, providing a level of insight at the single cell level. Furthermore, we also demonstrate that these models are applicable to the assessment of senescence *in vivo*, which remains a key challenge for the field. Therefore, we believe SAMPs has the potential to serve as a useful addition in the repertoire of senescence researchers, either as a first-pass tool or as part of the established senescence hallmarks.

## INTRODUCTION

Senescence induction is typically characterised through identification of multiple parallel markers including loss of cellular proliferation [1], increased tumour suppressor expression [2], presence of DNA damage markers [3], appearance of senescence-associated heterochromatin foci (SAHF) [4], increased senescence-associated β-galactosidase (SA-β-Gal) activity [5], acquisition of a senescence-associated secretory phenotype (SASP) [6], and the increased release of small extracellular vesicles [7]. However, senescence is a complex and heterogeneous phenomenon, varying between cell types and senescence inducing stimuli [8]. Consequently, no single marker is considered universal and experimentally a panel of markers must be optimised in each specific senescence setting to achieve a confident designation [9].

A conceptually similar process, considering multiple factors as part of a broader, holistic phenotype, is that employed in the field of image-based phenotypic profiling. Here, an extensive range of image-based measurements (referred to as “features”) are extracted from microscopy images and used to define cellular “morphological profiles” [10]. This approach refers not to the simple concept of cell “shape” but rather a range of nuclear and cellular measurements that are combined to reflect a complex overall cellular phenotype. This allows cells to be classified according to subtle nuances within the overall phenotype that are not obvious when only a specific feature is considered in isolation [10–12].

Interestingly, many of the features that comprise these morphological profiles have, in isolation, been proposed as potential markers of senescence. Cell size was one of the first hallmarks of senescence to be experimentally described, with senescent cells acquiring an enlarged, flattened cellular shape [13–15]. This has recently been mechanistically linked to senescence induction via a process of “cytoplasmic dilution”, whereby enlarged cell size contributes to homeostatic disruption, as cellular machinery is unable to scale production to provide for the growing cell’s requirements [16]. Furthermore, senescent cells have also been associated with enlarged nuclei and reduced DAPI staining intensity [17, 18]. Established senescence-associated changes in these features provide evidence that senescent cells may possess broader morphological profiles, distinct from those of their proliferating counterparts. Therefore, we hypothesised that image-based phenotypic profiling may provide a means by which to discriminate between senescent and proliferating cells.

Here, we have employed high-content analysis (HCA) to assess the morphological profiles of senescent cells induced via oncogene expression, paracrine SASP treatment, replicative exhaustion or DNA damage, according to 62 image-based features. Collectively, these features may be thought to comprise an overall “senescence-associated morphological profile (SAMP)” that allows senescent cells to be distinguished from their proliferating counterparts regardless of inducing stimuli or tissue of origin. Furthermore, we have also examined the value of dimensionality reduction via exploratory factor analysis, to understand whether distilling our features into a smaller number of conceptually related “latent factors” aids the biological interpretability of the generated profiles. Additionally, by utilising the power of HCA, we have assessed the single-cell heterogeneity within each model, reinforcing the growing appreciation that the process of senescence induction does not lead to the development of homogenous phenotypes, although the magnitude of this heterogeneity varies between models. Finally, we demonstrate that generation of such profiles is not limited to an *in vitro* setting by assessing the morphology of senescent cells within palbociclib treated human tumour xenografts. Overall, we have sought to provide a conceptual starting point for the assessment of senescent cells via phenotypic profiling, which we believe has the potential to significantly improve experimental workflows during the initial stages of senescence characterisation. A glossary of terms is included in Table 1, to provide clarity regarding the terminology used throughout.

**Table 1:** Glossary of terms.

**Term**	**Explanation**
**Condition**	Proliferation or senescence state within a particular senescence model
**Condition mean**	Mean of experimental replicates producing value for each proliferation or senescence condition
**Dimensionality reduction**	Data transformation that is representative of full data set but consists of fewer components
**Eigenvalue**	A value representing the variance of features accounted for by a latent factor
**Experiment mean**	Mean of individual wells across a single plate. Experimental replicates
**Exploratory factor analysis (EFA)**	Method of dimensionality reduction which aims to determine whether features are influenced by underlying latent factors
**Exploratory factor analysis model**	Mathematical model constructed via EFA composed of a defined number of latent factors and associated factor loading values
**Factor loadings**	Measure of the association between individual features and latent factors in an EFA model
**Features**	Image-based measurements of size, shape, intensity and spatial relationships. Examples include: cell area and nuclear intensity
**Representative profile**	Morphological profile comprising select features, representative of latent factor classifications in senescence
**Kaiser criterion**	Theoretical cut off for factor selection in EFA, retaining only factors with eigenvalues greater than 1
**Latent factor**	A cellular property that is not directly measured but alterations to which influence groups of correlated features
**Morphological profile**	Large collection of features providing comprehensive characterisation of cellular morphology
**Over factoring**	EFA model that comprises excess latent factors leading to the separation of closely related features
**Scree plot**	Plot displaying eigenvalues (y-axis) of potential latent factors (x-axis) in EFA
**Senescence model**	Method for inducing senescence in a specific cell type. Examples include: oncogene-induced senescence via oncogenic HRas expression in IMR90 fibroblasts
**Single target data**	Morphological profile data generated for individual cells
**Standard score normalisation**	Normalisation method based upon a population mean. Values correspond to standard deviations from the population mean
**Under factoring**	EFA model that comprises insufficient latent factors to separate distinct sets of features
**Well summary mean**	Mean of individual cells within a single well. Technical replicates
**Z-score normalisation**	Normalisation method based upon a predetermined control condition. Values correspond to standard deviations from the control mean

## RESULTS

### Establishing senescence models

**Figure 1:**
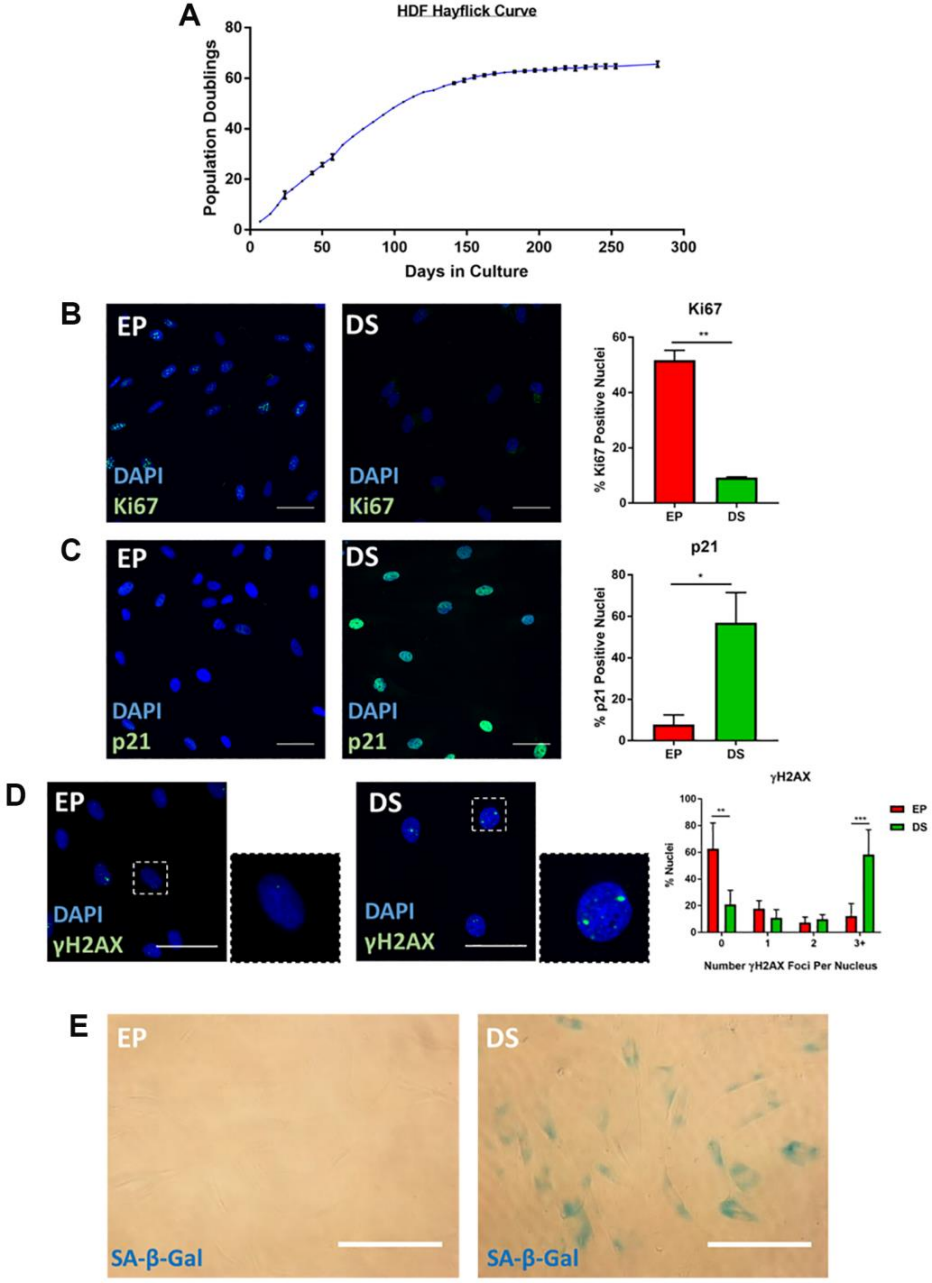
**Characterisation of senescence in human dermal fibroblasts (HDFs).** (**A**) Hayflick proliferation curve for human dermal fibroblasts (HDFs) from early proliferation (EP) to deep senescence (DS) through serial cell culture. *N* = 2–6. (**B**) Immunofluorescence staining of DAPI (blue) and Ki67 (green) in EP and DS HDFs. *N* = 2. Scale bar = 50 μm; (**C**) Immunofluorescence staining of DAPI (blue) and p21 (green) in EP and DS HDFs. *N* = 2. Scale bar = 50 μm (**D**) Immunofluorescence staining of DAPI (blue) and γ-H2AX foci (green) in EP and DS HDFs. *N* = 3. Scale bar = 50 μm. (**E**) Brightfield assessment of SA-β-Gal (blue) in EP and DS HDFs. *N* = 2. Scale bar = 100 μm.

A long-standing limitation within the senescence field is the lack of a universally accepted “marker” of senescence, with the collective assessment of a series of so called “hallmarks” widely accepted as best practice for a reliable classification [9, 19]. Our primary aim within this work, was to explore the intricacies of senescent cell morphology through phenotypic profiling, to understand whether such profiles could constitute a senescence hallmark themselves. An important first step in this process, was to characterise a range of established senescence models via conventional markers. We focused on exploring existing high-content images, as opposed to creating bespoke experiments for our phenotypic profiling, as we believe our approach could be readily implemented by other research groups wishing to extract profiling data from established datasets. This had the advantage of providing an inherent level of variability within the data, as it was generated by independent researchers for unrelated primary research aims. This approach also supported another of our aims: identifying a means of senescent cell classification that does not require resource intense optimisations and which may be applied in most research settings with microscopy capacity. The models employed here comprise four for which we have previously published characterisation data; oncogene-induced senescence (OIS) in IMR90s [20], paracrine senescence via conditioned media from OIS IMR90s [20], replicative senescence in adult human mammary fibroblasts (HMFs) [20, 21] and OIS in human mammary epithelial cells (HMECs) [22]. These image stacks were re-mined for this work, with previous senescence characterisation summarised in Supplementary Figure 1. Furthermore, we have also explored a model of replicative senescence in adult human dermal fibroblasts (HDFs), characterising canonical senescence markers including; loss of cellular proliferation by population doublings and Ki67 staining (Figure 1A and 1B) expression of the tumour suppressor p21 (Figure 1C), appearance of γH2AX DNA damage foci (Figure 1D) and increased SA-β-Gal activity (Figure 1E). As a supporting model, we also characterised UVB-induced senescence in HDFs via assessment of proliferation (Supplementary Figure 2A and 2B) and detection of DNA damage foci (Supplementary Figure 2C). Therefore, in each of the six models, cells were classified as either proliferating or senescent according to conventional senescence hallmarks.

### Phenotypic profiling of senescent cells – Z-Score profiling

**Figure 2:**
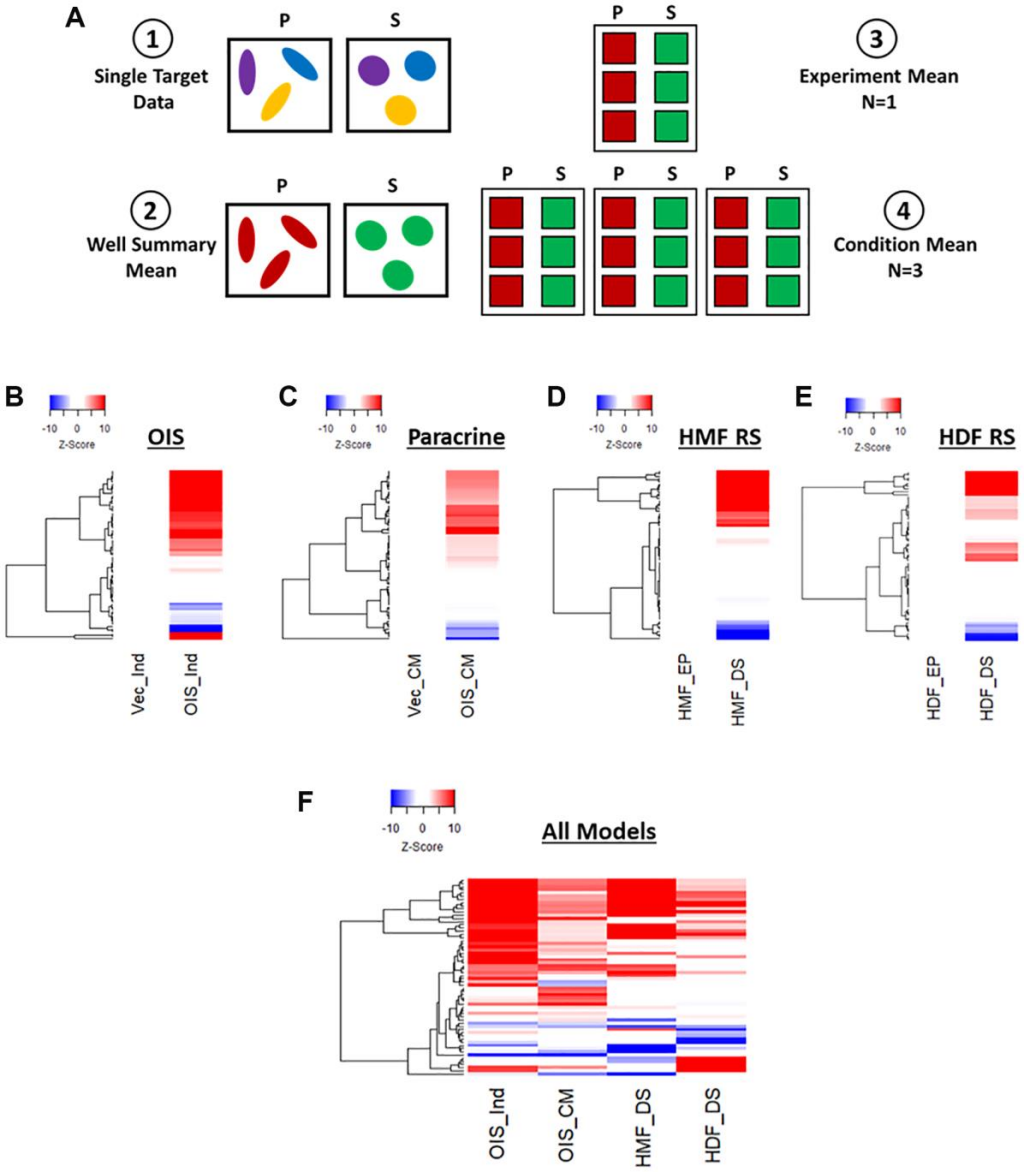
**Phenotypic profiling of senescence via Z-score profile generation.** (**A**) Schematic overview of data processing proceeding Z-score generation for each senescence model (P: proliferating condition, S: Senescence condition). (**B**–**E**) Z-score profile heatmaps for oncogene-induced senescence (OIS), paracrine senescence (Paracrine), HMF replicative senescence (HMF RS) and HDF replicative senescence (HDF RS) models. Y-axis comprises 62 morphological features (Red = positive modulation, Blue = negative modulation), White = no change). Proliferating conditions: vector induction (Vec_Ind), vector conditioned media (Vec_CM), HMF early proliferating (HMF_EP) and HDF early proliferating (HDF_EP). Senescence conditions: OIS induction (OIS_Ind), OIS conditioned media (OIS_CM), HMF deep senescence (HMF_DS), HDF deep senescence (HDF_DS). (**F**) Summary Z-score profile heat map of senescence conditions from each senescence model. Y-axis comprises 62 morphological features.

The initial stages of phenotypic profiling comprise the measurement of a large number of discrete image-based features, representing size, texture, spatial and intensity measurements from both nuclei and cells [10]. These are then combined to create a total profile for a particular treatment or cellular state. Here, we utilised two simple and widely used stains (DAPI and Cell Mask) to visualise the nuclear and cellular morphology of proliferating and senescent cells in each of our models. High-content image analysis was then performed, to generate measurements of 62 image-based features (Table 2) for every cell in each model. Means were then generated from both technical and experimental replicates (Figure 2A). Representative images from each model, along with nuclear and cellular masks upon which analysis was based, are summarised in Supplementary Figure 3.

**Table 2:** HCA phenotypic profiling features.

**1**	Nuclei Area	**32**	Nuclei Run Length Non uniformity
**2**	Nuclei Form Factor	**33**	Cells Area
**3**	Nuclei Elongation	**34**	Cells Form Factor
**4**	Nuclei Compactness	**35**	Cells Elongation
**5**	Nuclei Chord Ratio	**36**	Cells Compactness
**6**	Nuclei Gyration Radius	**37**	Cells Chord Ratio
**7**	Nuclei Displacement	**38**	Cells Gyration Radius
**8**	Nuclei Diameter	**39**	Cells Nuc/Cell Area
**9**	Nuclei Perimeter	**40**	Cells Diameter
**10**	Nuclei Intensity	**41**	Cells Perimeter
**11**	Nuclei Total Intensity	**42**	Cells Intensity (Cell)
**12**	Nuclei Intensity CV	**43**	Cells Intensity (Cyto)
**13**	Nuclei Light Flux	**44**	Cells Total Intensity (Cell)
**14**	Nuclei Intensity SD	**45**	Cells Total Intensity (Cyto)
**15**	Nuclei Major Axis	**46**	Cells Intensity CV (Cell)
**16**	Nuclei Minor Axis	**47**	Cells Intensity CV (Cyto)
**17**	Nuclei Spacing (SOI)	**48**	Cells Intensity Spreading
**18**	Nuclei Neighbor Count (SOI)	**49**	Cells Light Flux
**19**	Nuclei Spacing (MIN)	**50**	Cells Nuc/Cyto Intensity
**20**	Nuclei Neighbor Count (MIN)	**51**	Cells Intensity SD (Cell)
**21**	Nuclei Spacing (Gabriel)	**52**	Cells Intensity SD (Cyto)
**22**	Nuclei Neighbor Count (Gabriel)	**53**	Cells Major Axis
**23**	Nuclei Spacing (Lune)	**54**	Cells Minor Axis
**24**	Nuclei Neighbor Count (Lune)	**55**	Cells Skewness
**25**	Nuclei Skewness	**56**	Cells Kurtosis
**26**	Nuclei Kurtosis	**57**	Cells Energy
**27**	Nuclei Energy	**58**	Cells Entropy
**28**	Nuclei Entropy	**59**	Cells Grey Level Non Uniformity
**29**	Nuclei Grey Level Non Uniformity	**60**	Cells High GLRE
**30**	Nuclei High GLRE	**61**	Cells Low GLRE
**31**	Nuclei Low GLRE	**62**	Cells Run Length Non Uniformity

Abbreviations: SD: Standard Deviation; CV: Coefficient of Variance; GLRE: Grey Level Run Emphasis.

Next, Z-score normalisation was performed in order to compare features that would typically be on vastly different scales and, importantly, between senescence models [23]. This method centres the data to the mean value of the proliferating control in each model and scales based upon the standard deviation of the control. Thus, a Z-score of 1 represents a single standard deviation from the mean of the control condition. This was used within each model to produce a profile of Z-scores for each senescent condition. These were displayed as heatmaps, with positive and negative modulation from the control indicated as red or blue, respectively (Figure 2B–2E). In each of the senescence models, the senescent condition appeared distinct from the control in a number of features. However, the quantity and magnitude of these changes were highly variable between models. The OIS model produced the most striking profile, with potent change from the control condition in 49 out of 62 features. The paracrine senescence profile appeared to have less potent changes, but featured significant alterations in 50 out of 62 features. Both replicative senescence models produced more subtle profiles, with significant alterations in 38 out of 54 features for the senescent HMFs (8 features could not be assessed due to use of an older generation of microscope) and 37 out of 62 features in the senescent HDFs, albeit where changes did occur, they did so potently. This is visualised in the summary heat map (Figure 2F). Of note, the direction of modulation of many features appears consistent between models, opening the possibility that the profiles reflect common senescence-associated changes. However, the Z-score method of normalisation has the disadvantage that the profiles produced are relative to the control condition, thereby masking the “profile” of the proliferating cells. To overcome this limitation, we revised our data processing steps to generate meaningful profiles for both proliferating and senescent conditions.

### Phenotypic profiling of senescent cells – standard score profiling

**Figure 3:**
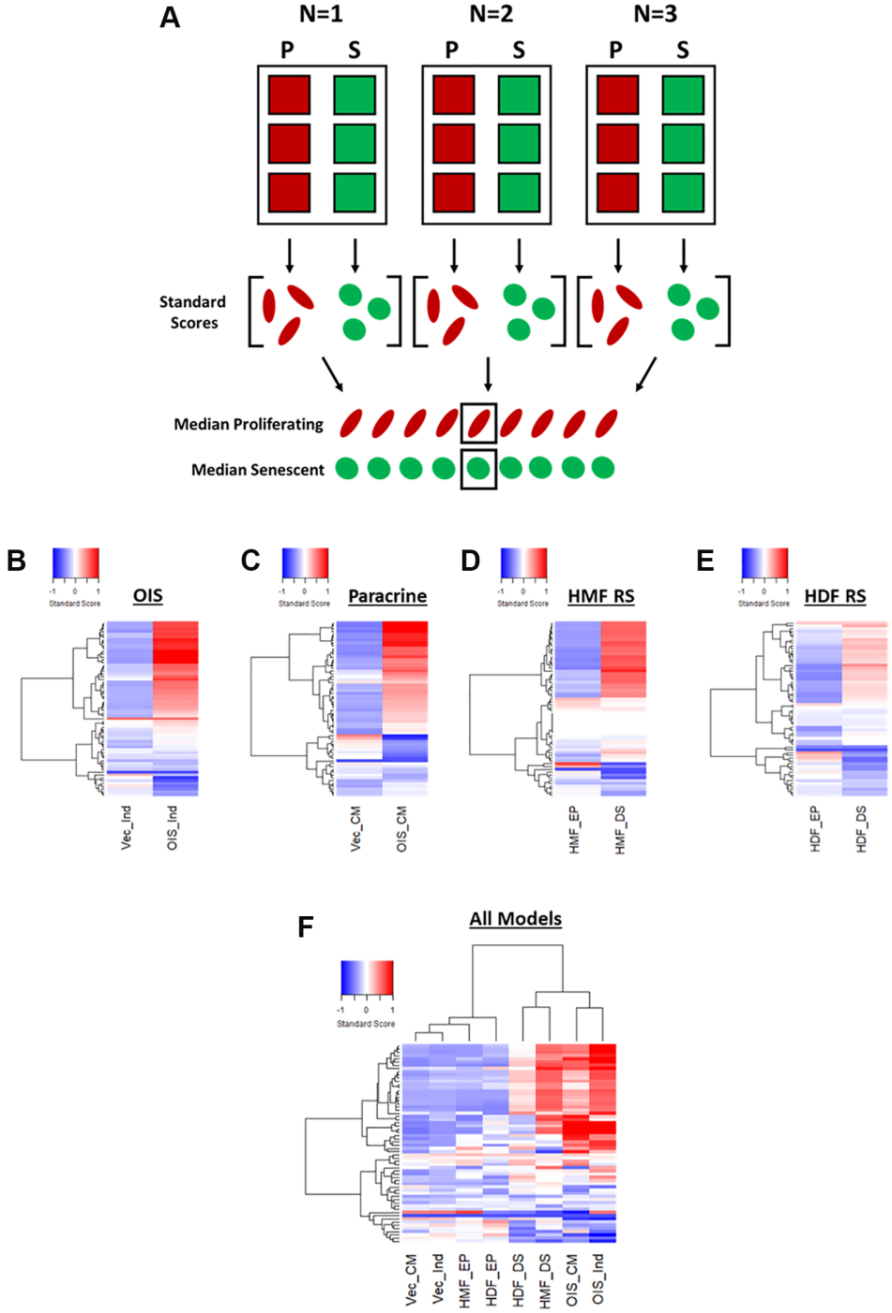
**Phenotypic profiling of senescence via standard score profile generation.** (**A**) Schematic overview of data processing proceeding standard-score generation for each senescence model (P: proliferating condition, S: Senescence condition). (**B**–**E**) Standard-score profile heatmaps for oncogene-induced senescence (OIS), paracrine senescence (Paracrine), HMF replicative senescence (HMF RS) and HDF replicative senescence (HDF RS) models. Y-axis comprises 62 morphological features (Red = positive modulation, Blue = negative modulation), White = no change). Proliferating conditions: vector induction (Vec_Ind), vector conditioned media (Vec_CM), HMF early proliferating (HMF_EP) and HDF early proliferating (HDF_EP). Senescence conditions: OIS induction (OIS_Ind), OIS conditioned media (OIS_CM), HMF deep senescence (HMF_DS), HDF deep senescence (HDF_DS). (**F**) Summary standard-score profile heat map and hierarchical clustering of all proliferating and senescence conditions. Y-axis comprises 62 morphological features.

In order to provide a means of data scaling to facilitate comparisons between features and senescence models, the single target data was used to produce “standard scores” [10]. These utilise the mean and standard deviation values from the combined proliferating and senescent single target data within each model, to produce a data set comprising standard scores for each of the 62 features, for every cell in each model (Figure 3A). Median scores for each proliferating and senescent condition were then used to produce heatmap profiles. These supported the observations made using Z-scores, in that the senescent condition in each model was distinct from its proliferating counterpart (Figure 3B–3E). However, whereas some features were indistinguishable from the proliferating control by Z-scores, the standard scores demonstrated modulation in the vast majority of features in each model between the proliferating and senescent states. Similar observations to those of Z-score normalisation were also made in terms of the magnitude of change between models, with the OIS model producing the most potent phenotype and the replicatively senescent HDFs a more subtle one. When all senescence models were compared, hierarchical clustering demonstrated a clear distinction between the senescent conditions and those of the proliferating controls (Figure 3F).

Therefore, it appears that phenotypic profiling may be used to clearly distinguish between senescent and proliferating cells based upon the development of a “senescent-associated morphological profile”. This profile is present within each senescence model but varies in terms of magnitude. Furthermore, these complex profiles seem to consist of both a “core signature” of features that change commonly within senescence, as well a number of idiosyncratic features that reflect the established heterogeneity between inducers of senescence. Alternatively, this could be the result of the different tissues from which the cells were derived or, more likely, a combination of the two. In order to explore this further, we utilised a model of DNA damage-induced senescence in the HDFs via UVB treatment (Supplementary Figure 2A–2C). This led to more potent profiles (both Z-score and standard score) than observed in RS HDFs, supporting the observation from the IMR90s that different inducers of senescence in the same cells produce distinct phenotypic profiles (Supplementary Figure 2D and 2E). In order to emphasise this further, we then applied our analysis pipeline to OIS HMECs, as to this point we had only evaluated fibroblasts (Supplementary Figure 4). As with the IMR90 model, the HMECs were associated with strikingly potent profiles in the OIS condition, both demonstrating the utility of our analysis pipeline in other cell types and further supporting the utility of phenotypic profiling as a powerful tool for senescence characterisation. Importantly, the utilisation of standard scores provided significant benefit over Z-scores, in understanding the nuance between each phenotypic profile. This included the ability to interpret the profile of features in the control condition, allowing comparison of these between models.

### Phenotypic profile generation by exploratory factor analysis

#### EFA model development

Whilst the standard score profiles facilitated the distinction between proliferating and senescent cells in a number of models, the high dimensionality of the data made biological interpretability difficult, as well as any comparison to established senescence-associated features. To overcome this, we adopted a strategy similar to that of Young et al., utilising exploratory factor analysis (EFA) as a method of dimensionality reduction [11]. EFA seeks to determine whether the changes observed in measured features occur through alterations to so called “latent factors” [24, 25]. In the context of HCA, these are assumed to be cellular properties which, whilst not directly assessed, are of potentially more biological relevance than the measured features themselves [11]. Therefore, EFA has the potential to complement HCA by identifying the underlying relationships between features, allowing them to be considered collectively. Here, we utilised EFA to understand whether a select group of biologically interpretable latent factors could provide insight into the morphological phenotypes of senescent cells.

**Figure 4:**
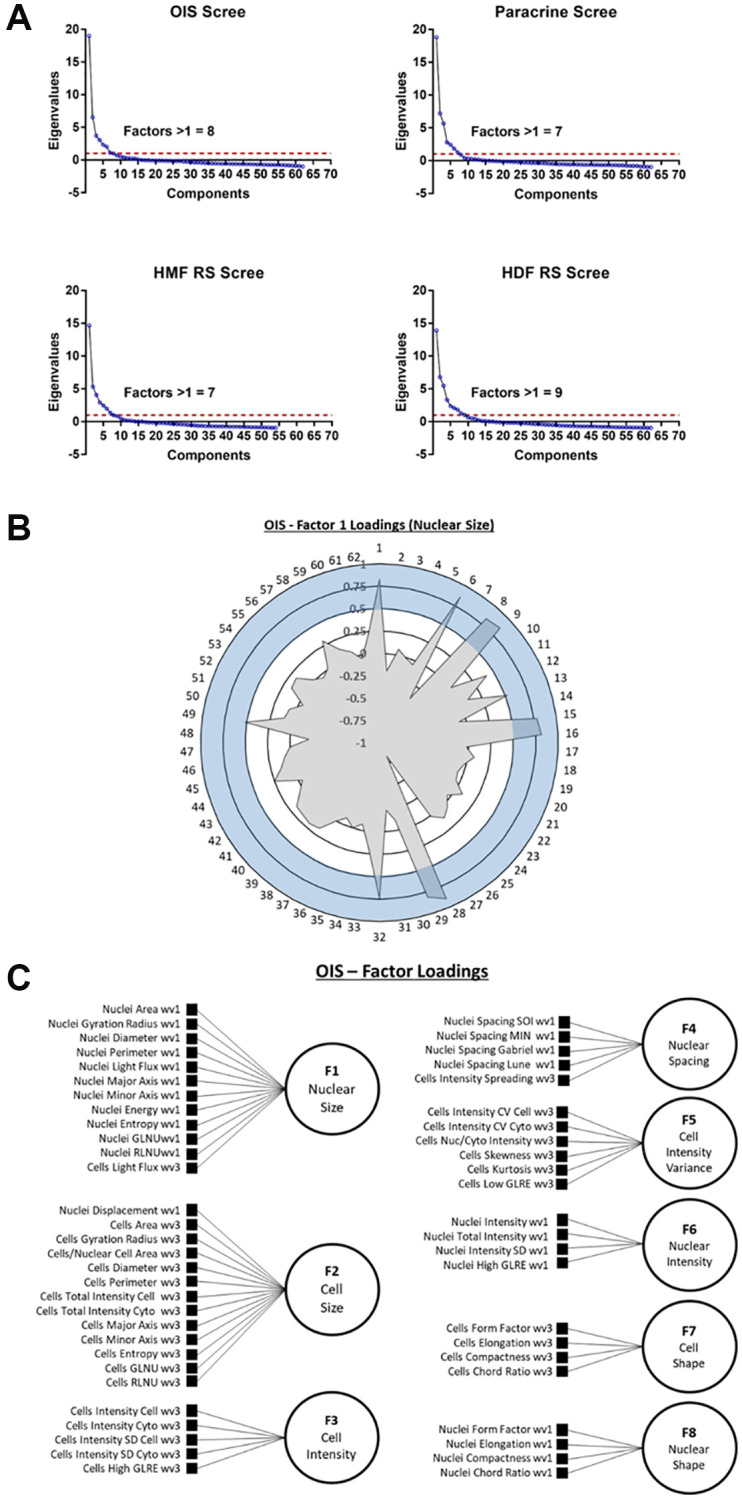
**Exploratory factor analysis (EFA).** (**A**) Scree plots for oncogene-induced senescence (OIS), paracrine senescence (Paracrine), HMF replicative senescence (HMF RS) and HDF replicative senescence (HDF RS) models. Red line indicates eigenvalue = 1. (**B**) Polar plot of factor loading values for factor 1 from OIS EFA model (designated Nuclear Size). 1–62 refer to features (Table 2). Blue shaded area indicates factor loading threshold of 0.5. (**C**) Factor loading diagram for OIS EFA model with factor designations. Equivalent diagrams for other senescence models are found in Supplementary Figure 3).

First, the number of latent factors underpinning the full list of 62 features needed to be established. Initially, data from each individual senescence model was used in isolation, to construct four unique EFA models. Scree plots were produced in each instance to determine the number of latent factors. These represent the total variance accounted for by each potential latent factor as an eigenvalue, with the total number of possible latent factors equal to the total number of features (i.e., 62). By employing the widely accepted Kaiser criterion, only factors that had an eigenvalue greater than 1 were retained for factor analysis within each model [26, 27]. This resulted in the following totals of latent factors: OIS induction (8), paracrine senescence (7), RS HMF (7) and RS HDF (9) (Figure 4A). Next, factor loadings were determined for each latent factor. This refers to how closely individual features are associated with each latent factor, and can be considered similar to standard regression coefficients. A stringent loading threshold of 0.5 was selected, so that only features that loaded with a value greater than this were included in each factor. For illustrative purposes, factor loadings for a single factor from the OIS induction EFA model, has been visualised as a polar plot (Figure 4B). Next, the feature composition of individual latent factors in each EFA model was assessed in order to assign potential factor classifications. Interestingly, in each EFA model, the latent factors could be broadly classified according to the following designations 1) Nuclear Size, 2) Cell Size, 3) Cell Intensity, 4) Nuclear Intensity, 5) Nuclear Spacing, 6) Nuclear Shape, 7) Cell Shape 8) Cell Intensity (variance) and 9) Nuclear Intensity (variance), based on the composite features. As EFA represents an iterative tool for investigating relationships within data, as opposed to one applying inflexible statistical cut offs, the total number of latent factors was adjusted to 8 in each EFA model at this point, as initial factor loadings indicated “under-“ and “over-factoring” in the models utilising 7 and 9 latent factors, respectively (see Table 1). By employing this iterative approach, EFA models were constructed for each senescence model, with 7 common factor designations, along with one of either Cell Intensity (variance) or Nuclear Intensity (variance). In order to visualise the composition of factors within each EFA model, as well as the factor classifications themselves, schematic representations were produced (Figure 4C and Supplementary Figure 5).

#### EFA model analysis

**Figure 5:**
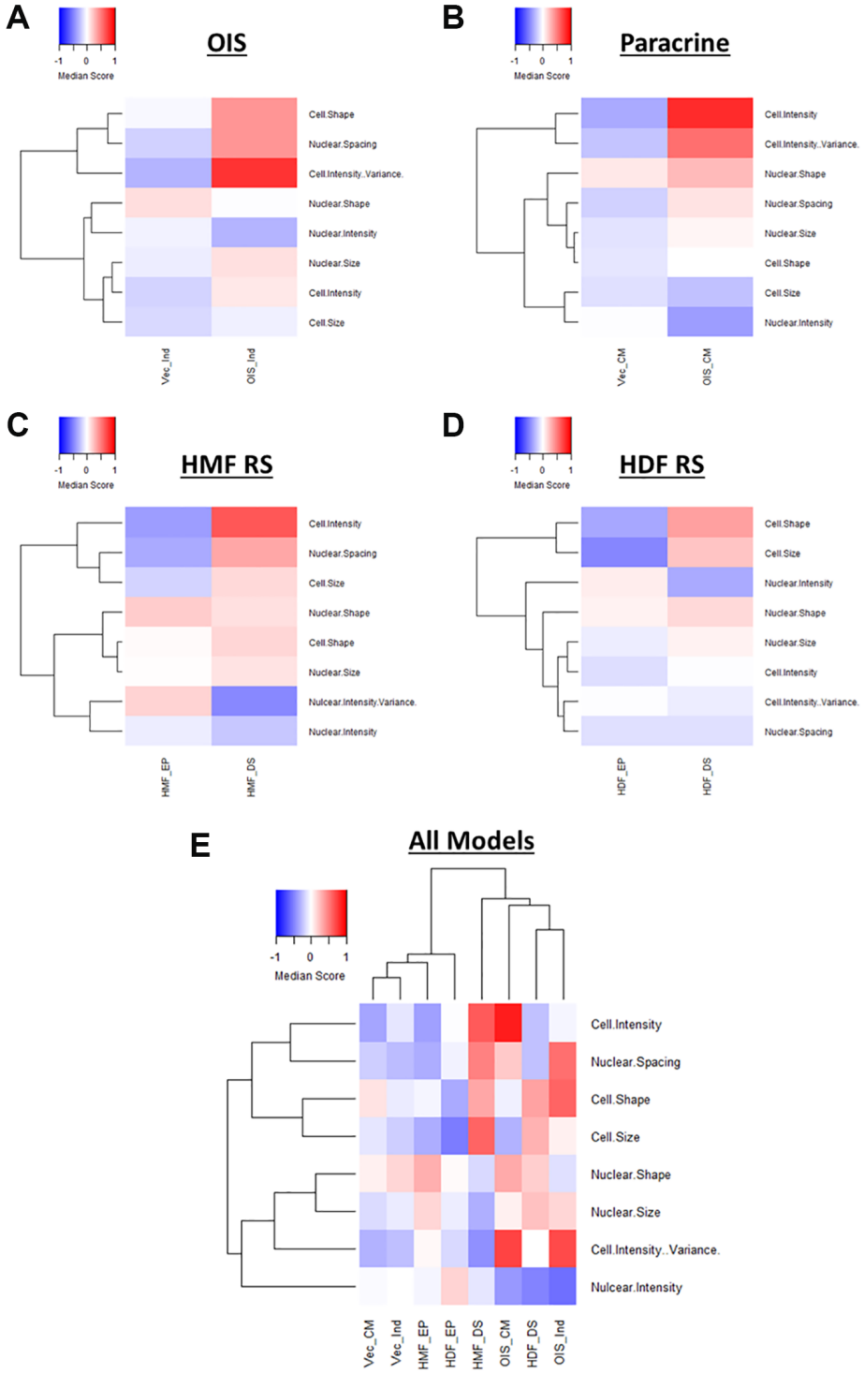
**Factor score profiles following EFA.** (**A**–**D**) Factor score profile heatmaps for oncogene-induced senescence (OIS), paracrine senescence (Paracrine), HMF replicative senescence (HMF RS) and HDF replicative senescence (HDF RS) models. Y-axis comprises scores for 8 latent factors determined by EFA (Red = positive modulation, Blue = negative modulation), White = no change). Proliferating conditions: vector induction (Vec_Ind), vector conditioned media (Vec_CM), HMF early proliferating (HMF_EP) and HDF early proliferating (HDF_EP). Senescence conditions: OIS induction (OIS_Ind), OIS conditioned media (OIS_CM), HMF deep senescence (HMF_DS), HDF deep senescence (HDF_DS). (**E**) Summary factor score profile heat map and hierarchical clustering of all proliferating and senescence conditions. Y-axis comprises scores for 8 latent factors determined by EFA.

Following EFA model development, factor scores were extracted for each cell within each senescence model and median score profiles generated (Figure 5A–5D). Once again, these clearly demonstrated a distinction between the proliferating and senescent condition within each senescence model. However, they also provided insight into the relative importance of each latent factor in determining this distinction. For example, Cell Shape appears to contribute less to the overall phenotype of the paracrine senescent cells, as opposed to the other three models. Conversely, Cell Intensity changes dramatically in the paracrine senescent cells but much less so in the RS HMFs. Therefore, EFA aids the assessment of phenotypic profiles by reducing the overall complexity and directing attention to specific interpretable factors within each model. It is important to note that the factors can only cautiously be compared between models in this way, as whilst similar, the underlying features which contribute to each latent factor (as well as the relative factor loadings) are variable. In order to overcome this, EFA was performed using the combined single target data from all senescence models. Scree plot and eigenvalue assessment suggested that a model comprising 8 latent factors should be constructed, supporting the earlier iterative adjustment to this latent factor threshold (Supplementary Figure 6A). Furthermore, each factor could be identified according to the designations previously established (Supplementary Figure 6B and 6C). Factor scores were again used to construct profiles for each condition, which could now be directly compared, having been based on the same EFA model (Figure 5E). As with the standard score profiles, hierarchical clustering demonstrated a clear distinction between the senescent and proliferating conditions, albeit with the development of highly heterogeneous senescence-associated factor profiles (Figure 5E). This demonstrated that EFA does not preclude phenotypic profiling-based senescence identification despite the large reduction in profile components.

The primary aim of EFA was to identify relationships between features and reduce the complexity of the phenotypic profiles in order to aid interpretation. Whilst we have taken an unbiased and systematic approach, the consistency of latent factor designations between senescence models suggests that extraction of a set of select features could be used as a more straightforward overview of the broader profiles (Supplementary Figure 7A). These representative profiles are less complex to produce and maintain distinction between senescent and proliferating cells both within individual models (Supplementary Figure 7B–7E) and when comparing all models (Supplementary Figure 7F). Such profiles could serve as more accessible (though still valid) representation of senescence-associated morphological profiles, particularly as the majority of the composite features have established literature precedent. Therefore, EFA complements the phenotypic profiling of senescent cells by providing several options for reduced profile complexity and interpretability. However, the reduced factor profiles also limit the potential for more nuanced comparison between models, which we consider a key advantage of the more complex phenotypic profiles. Therefore, recognising a major strength of the initial approach, the highly dimensional nature of the data produced by HCA, we sought to explore the heterogeneity of profiles generated on a single target level.

### Exploring the heterogeneity of senescence-associated morphological phenotypes

**Figure 6:**
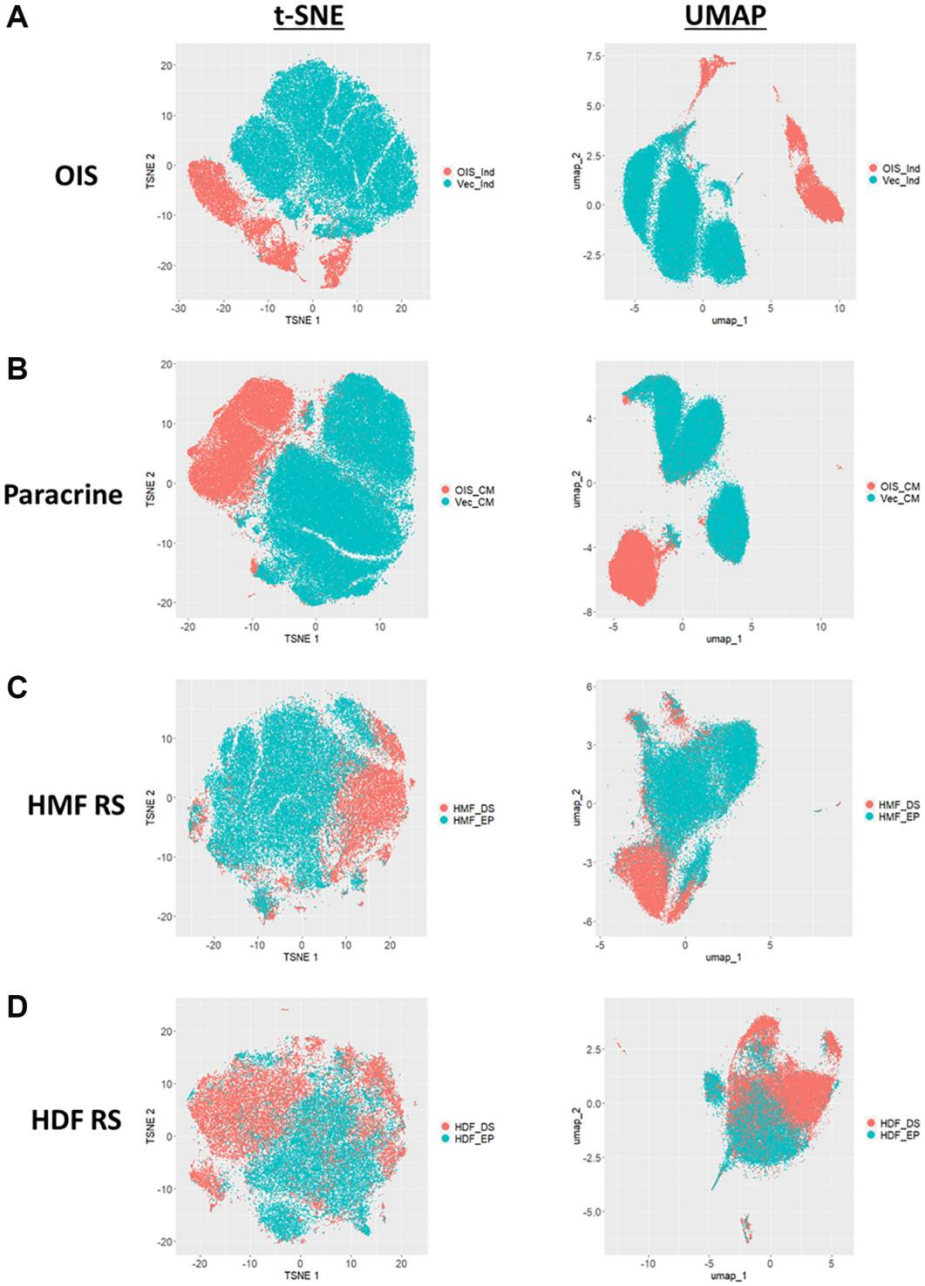
**t-SNE and UMAP profiles for single target morphological profiles.** (**A**–**D**) t-SNE and UMAP plots for oncogene-induced senescence (OIS), paracrine senescence (Paracrine), HMF replicative senescence (HMF RS) and HDF replicative senescence (HDF RS) models. Proliferating conditions (Blue): vector induction (Vec_Ind), vector conditioned media (Vec_CM), HMF early proliferating (HMF_EP) and HDF early proliferating (HDF_EP). Senescence conditions (Orange): OIS induction (OIS_Ind), OIS conditioned media (OIS_CM), HMF deep senescence (HMF_DS), HDF deep senescence (HDF_DS).

Both the Z-score and standard score profiles demonstrated that in each model of senescence, a distinct senescence-associated morphological phenotype could be observed. However, each model was characterised by a degree of heterogeneity, both in terms of the magnitude and direction of change in the features observed. We classify this as inter-model heterogeneity, likely representing the distinct pathways engaged in the development of senescence via different inducing stimuli. Another key consideration developing within the field is that of intra-model heterogeneity, the development of distinct populations of senescent cells within a single model. The most well described example of this phenomenon is that of NOTCH1 mediated juxtacrine secondary senescence during OIS induction [28, 29]. Therefore, one would anticipate a degree of heterogeneity within each model, as different populations of cells are induced by distinct stimuli. To explore this, the single target standard scores were explored in a manner similar to that more commonly seen during single-cell RNA sequencing experiments. T-distributed stochastic neighbour embedding (t-SNE) and Uniform Manifold Approximation and Projection (UMAP) algorithms were employed in order to represent the single target data in an interpretable form. Within these plots, each point represents the high-content, 62-feature profile of a single cell (Figure 6A–6D). Strikingly, in each model, regardless of clustering method, the population of senescent cells was distinct from that of the proliferating control. In the OIS model, two well-defined populations of senescent cells could be observed by both t-SNE and UMAP, aligning with the concept that OIS has both a primary, oncogene-driven component and a second, juxtacrine component, although comprehensive characterisation of NOTCH1 expression would be required to confirm this. The paracrine senescence model had a far more homogenous population of senescent cells, possibly due to the nature of the inducing stimuli being treatment with a SASP comprising a consistent composition. Interestingly, whilst primary senescent clusters were reasonably distinct in both replicative senescence models, this was less apparent than seen in the IMR90 models. This could be attributed to previous observations regarding the complex set of stimuli underpinning replicative senescence, including a degree of paracrine senescence, mitochondrial dysfunction, oxidative damage and telomere shortening within the total population [30]. Furthermore, as evident from the HDF Hayflick curve (Figure 1A), replicative senescence is a far more gradual process than seen in the OIS and paracrine forms of premature senescence. Thus, a greater degree of heterogeneity might be expected, given some cells would likely have reached senescence potentially weeks before others. Overall, this data aims to serve as an alternative visualisation of the morphology profiles, demonstrating variability in the phenotypes at the single cell level, which is not achievable when represented as a heatmap. We suggest that HCA shows good potential in facilitating the opportunity to explore potential heterogeneity present both between and within senescent models, as well as reinforcing our earlier observation that senescent cells are associated with distinct and complex morphological phenotypes.

### Applying phenotypic profiling to senescence *in vivo*

**Figure 7:**
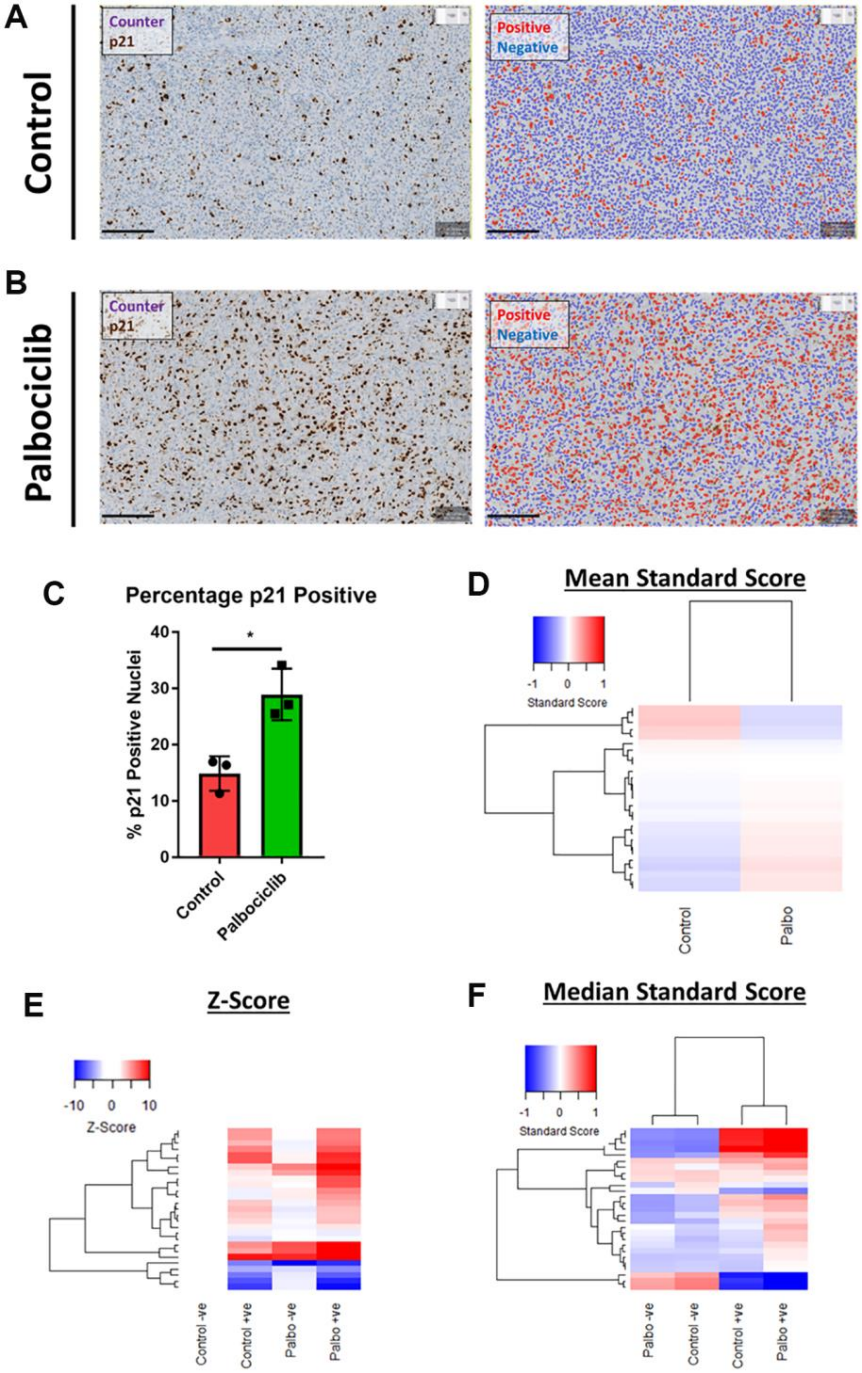
**Assessment of senescence morphology in palbociclib treated human tumour xenografts.** (**A** and **B**) Analysis mask generation to define p21 positive (red) and negative (blue) nuclei in control and palbociclib treated xenografts. Blue stain = Counterstain, Brown stain = p21. Scale bar = 200 μm. (**C**) Percentage p21 positivity of nuclei in control and palbociclib treated xenografts. (**D**) Mean standard score profiles of control and palbociclib samples. Y-axis comprises 27 morphological features (Red = positive modulation, Blue = negative modulation), White = no change). (**E** and **F**) Z-score and standard score profiles of p21 positive and negative cells in control and palbociclib treated xenografts. Y-axis comprises 27 morphological features (Red = positive modulation, Blue = negative modulation), White = no change). *N* = 3.

Despite the emergence of a variety of hallmarks *in vitro*, the availability of reliable *in vivo* markers of senescence remains a challenge for the field. In order to understand whether our observation of *in vitro* senescence-associated morphological profiles were also detectable *in vivo*, we assessed the morphology of individual cells within human SK-MEL-103 xenograft tumours following treatment with palbociclib. We utilised p21 staining to define senescent cells within each treatment and confirmed that the percentage of p21 positive (i.e. senescent) cells increased following palbociclib treatment (Figure 7A–7C). When all cells in each treatment condition were assessed collectively, a subtle yet distinct phenotype comprising 27 morphological features (Table 3) distinguished the palbociclib condition from the control, suggesting such profiles could have utility as a prognostic marker of senescence *in vivo*, or form the basis for a classification tool (Figure 7D). More strikingly, when p21 positive and negative cells were considered separately, within each treatment condition a potent difference in morphological profile was apparent between the senescent and non-senescent cells (via both Z-score and standard score; Figure 7E and 7F, respectively). Hierarchical clustering of standard scores confirmed this distinction, suggesting that the alterations in morphological profiles we observe between senescent and non-senescent cells is not limited to *in vitro* settings. Importantly we utilised open source software to perform this analysis (QuPath), demonstrating that this methodology could be widely applied within the senescence research community [31].

**Table 3:** *In vivo* phenotypic profiling features.

**1**	Nucleus: Area	**15**	Cell: Circularity
**2**	Nucleus: Perimeter	**16**	Cell: Max Caliper
**3**	Nucleus: Circularity	**17**	Cell: Min Caliper
**4**	Nucleus: Max Caliper	**18**	Cell: Eccentricity
**5**	Nucleus: Min Caliper	**19**	Cell: Counterstain OD Mean
**6**	Nucleus: Eccentricity	**20**	Cell: Counterstain OD SD
**7**	Nucleus: Counterstain OD Mean	**21**	Cell: Counterstain OD Max
**8**	Nucleus: Counterstain OD Sum	**22**	Cell: Counterstain OD Min
**9**	Nucleus: Counterstain OD SD	**23**	Cytoplasm: Counterstain OD Mean
**10**	Nucleus: Counterstain OD Max	**24**	Cytoplasm: Counterstain OD SD
**11**	Nucleus: Counterstain OD Min	**25**	Cytoplasm: Counterstain OD Max
**12**	Nucleus: Counterstain OD Range	**26**	Cytoplasm: Counterstain OD Min
**13**	Cell: Area	**27**	Nucleus/Cell area ratio
**14**	Cell: Perimeter		

Abbreviations: SD: Standard Deviation; OD: Optical Density.

## DISCUSSION

Senescence represents a diverse set of terminal cell fates that display a significant degree of heterogeneity depending on the stimulus to which a cell has been exposed. In our view, this makes the search for a single universal marker of senescence unlikely, particularly when we consider that even within a single model of senescence induction, individual cells may be driven to arrest through entirely different mechanisms. However, many of these inducing stimuli engage common pathways, which has allowed senescence identification to take place through the characterisation of common “hallmarks”. Experimentally this process is both practically laborious and resource intensive, with a high level of optimisation required in each specific senescence setting. Consequently, it has recently been proposed that a multi-stage workflow be employed in the classification of senescent cells, starting with higher-throughput assays, such as SA-β-Gal, with more focused follow up assays to determine senescence “sub-classifications” [8, 32]. In this work we have demonstrated that adoption of a phenotypic profiling-based approach, in combination with HCA, provides a significant level of insight into the development of senescence in cells from a variety of tissues, induced by multiple stimuli. Whilst the changes in cell morphology represent one of the earliest reported markers of senescence [13, 14], this has previously relied on the characterisation of single morphological features employed in isolation [33]. Here, we demonstrate that HCA provides an additional layer of sophistication, facilitating a comprehensive assessment of cellular morphology through complex and multifaceted profiles. Importantly, the protocol used to achieve this required only the use of two widely available and inexpensive cell dyes and, did not require significant optimisation. Furthermore, whilst we have utilised a dedicated HCA image analysis software, comparable workflows have been successfully implemented previously with alternative, open-source software packages, albeit not in the context of senescence [11, 12]. This, coupled with our re-mining based approach, gives us confidence that generation of SAMP profiles is readily achievable in most research settings. Therefore, as a methodology, it has the potential to fulfil the field’s growing appetite for a reliable “first-pass” method of senescence characterisation that would be of particular use in the context of screening [32]. It is important to emphasise that we have not sought to develop such a screening tool for the identification of senescence in this work, but rather to explore the inherent biology of the morphological features associated with senescent cells. These features have been aggregated into phenotypic profiles and demonstrate a striking distinction between senescent and actively cycling cells. The profiles have also allowed us to demonstrate the existence of a “core-signature” of senescence-associated features, as well as those that underpin both the inter- and intra- model heterogeneity widely reported in the context of senescence. Furthermore, the strength of HCA lies in the generation of high dimensionality data, which provides scope for nuance and subtlety in the profiles generated, thus expanding the complexity of potential comparisons between models. We also demonstrate that applying the same analysis principles allows the distinction of senescent cells *in vivo*, which remains a key challenge within the field. Whilst, we are hesitant to introduce a new acronym into the expanding lexicon of senescence related terms, we colloquially refer to these senescence-associated morphological profiles as SAMPs. This is in part, due to the conceptual overlap with that of the SASP, in as much as both are multifaceted profiles that appear to be common following senescent induction, albeit comprising significant heterogeneity in terms of composition and potency. Interestingly, in the case of both the SASP and SAMP, OIS appears to be associated with particularly potent profiles, perhaps reflecting the supra-physiological nature of that particular model [2, 6].

Overall, we believe that we have demonstrated that phenotypic profiling has both scope and potential to aid researchers in the classification of senescent cells. There is growing interest in the development of screening tools for the identification of senescence and analysis of the SAMP could form the basis of such a classification system, particularly in combination with machine learning algorithms [34, 35]. Furthermore, whilst we have adopted a protocol that makes use of only nuclear and cellular features, the incorporation of dyes for other cellular compartments (such as mitochondria and the endoplasmic reticulum) could provide an even greater level of insight into the complexity of cellular responses following senescence induction, as has been successfully utilised elsewhere with the concept of “cell painting” [36]. Ultimately, a key benefit of phenotypic profiling is its potential for customisation depending on the requirements of the researcher, with additional layers of complexity incorporated with relative ease. Whilst we have demonstrated that senescence is associated with the development of a SAMP in multiple contexts, we have no doubt that additional complexity will emerge as the number of cell types and senescence models investigated in this manner increases. Therefore, it is our conclusion that the SAMP profiles generated via HCA and phenotypic profiling represent a marker of senescence that may be added to the growing toolbox of senescence hallmarks.

## METHODS

### Cell culture and reagents

Unless indicated, all reagents were sourced from Sigma, UK. IMR90 ER: STOP (vector) and ER:RAS (OIS) fibroblasts were produced as described previously [20, 37] and were kindly gifted by Juan Carlos Acosta (MRC Institute of Genetics & Molecular Medicine, Edinburgh). All cells were maintained in Dulbecco’s Modified Eagles Medium (DMEM; Life Technologies, UK) with additional 10% foetal bovine serum (FBS, Labtech.com, UK) and 2mM L-glutamine (DMEM; Life Technologies, UK). Human mammary fibroblasts (HMFs) were further supplemented with 10 μg/ml bovine pancreas insulin and were kindly provided by Martha Stampfer (Lawrence Berkeley National Laboratory, Berkeley). Tamoxifen inducible ER: RAS (OIS) telomerase-immortalised human mammary epithelial cells (HMEC) were cultured as previously described [22]. Human dermal fibroblasts (HDFs) were anonymously donated by healthy patients (LREC No. 09/HO704/69). Cells were cultured at 37°C/5% CO_2_ without the presence of antibiotics. All experimental end points utilised cells at sub-confluent densities.

### Antibodies

The following antibodies were used in for immunofluorescence staining: p21 (2947, Cell Signalling, UK; 1:500), Ki67 (NCL-Ki67p, Novocastra, Cell Signalling, UK; 1:1,000), γH2AX (05-636, Upstate Cell Signalling Technologies; 1:2,000), 53BP1 (A300-273A, Bethyl laboratories, 1:200), goat anti-mouse-Alexa Fluor 488 (A32723, Thermo Fisher, UK; 1:500) and donkey anti-rabbit-Alexa Fluor 546 (A10040, Thermo Fisher, UK; 1:500). Immunohistochemistry for p21 was performed using mouse anti-human p21 (M7202, Agilent Dako, US, 1:100), recombinant IgG1+IgG2+IgG3 bridging antibody (ab1334569, Abcam, UK, 1:500) and OmniMap anti-Rb HRP secondary antibody (5269679001, Roche, according to the manufacturer’s instructions).”

### Senescence induction

Senescence induction was performed as described previously for both the OIS, paracrine senescence and HMF RS models [20–22]. HDFs were serially cultured to replicative senescence (>200 days) and designated as having reached deep senescence (DS) at passage 39 plus an additional 3 weeks (~280 days). For phenotypic profiling by HCA, early proliferating (EP) HDFs were seeded at 4,000 cells/cm^2^, whilst DS HDFs were seeded at 10,000 cells/cm^2^. For UVB-induced senescence, HDFs were seeded at 5,000 cells/cm^2^ in 10 cm dishes (Starlabs, UK), with the control seeded at 4,000 cells/cm^2^. Cells were incubated for 2 days in standard culture medium, and UV-treated for 5 consecutive days at a dose of 0 mJ/cm^2^ (Control) or 6 mJ/cm^2^ (UVB) (UV-B lamp G8T5E, Sankyo-Denki). During UVB treatment, normal culture medium was replaced with D-PBS (0.9 mM CaCl_2_, 0.5 mM MgCl_2_-6H_2_O; Life Technologies, UK). The cells were the incubated in standard growth medium for a further 2 days before fixation and characterisation.

### Immunofluorescence staining and high-content analysis (HCA) microscopy

Immunofluorescence staining and HCA microscopy was performed as described previously [20, 21]. Briefly, following fixation with 3.7% paraformaldehyde, cells were permeabilised with 0.1% Triton X-100 and stained with diamidino-2 phenylindole (DAPI) (Sigma UK, D8417, 1:1,000) and HCS Cell Mask Deep Red (Thermo-Fisher UK, C10046, 1:50,000 – 1:100,000) for 2 hours at room temperature. Cells were then imaged using an IN Cell 2200 automatic microscope (IMR90s and HDFs) or an IN Cell 1000 automatic microscope (HMFs). For senescence marker assessment, cells were blocked for 30 minutes with PBS/BSA following permeabilsation and stained with primary antibody diluted in PBS/BSA for 2 h at room temperature or overnight at 4°C. A PBS/BSA wash step was then used, followed by secondary antibody staining for 2 h at room temperature.

### High-content analysis (HCA) and data processing

Images from all senescence models were analysed via InCarta image analysis software (Cytiva). DAPI staining was used to visualise nuclei, whilst Cell Mask staining allowed cellular visualisation. InCarta detection protocols were developed in order to generate nuclear and cellular image masks, upon which data analysis was performed (Supplementary Figure 3). These were consistent within individual experiments, with a level of fine tuning between replicates to ensure optimal mask generation. Image masks were then analysed according to pre-built features (termed measures in the InCarta software), with all features selected apart from those that assess “background” intensities, centre of gravity co-ordinates and major axis angles. These were excluded as they are principally tools for assessing imaging consistency as opposed to biologically profiling cells as opposed to specific cellular properties. This leads to a final list of 62 features, which formed the basis of the phenotypic profiles (Table 2). It is important to note that as we used bespoke HCA software, this is a lower number of total features than is sometimes used for phenotypic profiling, due to a lack of redundancy amongst features [12]. However, phenotypic profiling has been successfully employed with a feature count as low as 35, leading us to believe our profiles to be sufficiently comprehensive [11].

Following HCA, data normalisation was required in order to facilitate comparison between features on different scales. This was initially performed via Z-score normalisation compared to a proliferating control condition using the following equation, as used elsewhere [20, 21, 23]. Z-Score **=** mean value of three independent experiments for senescent experimental condition **–** mean value of three independent experiments for control condition / Standard Deviation (SD) of control condition. Z-score profiles were displayed as heat maps, where significant positive or negative change was defined as a Z-score greater than 1.96 and visualised as either red or blue, respectively. Subsequently, single target data was used to generate standard score feature profiles for individual cells from each condition in all models according to the following equation. Standard Score = value of an individual cell – mean value of all targets (proliferating and senescent) / Standard Deviation (SD) of all targets (proliferating and senescent). Standard scores were generated within experimental replicates. Standard scores from all three replicates per condition were then combined and median profiles calculated (to reduce the influence of outliers). These were presented as heat maps, with positive modulation of features represented as red and negative modulation of features represented as blue.

It is important to note that HMF and HMEC image acquisition was performed via an older generation of microscope (INCA 1000), which produces rectangular images as opposed to the square images produced by the INCA2200 that all other models were assessed using. This means that 8 features (Features 33,34,35,36,66,67,68 and 69 – Table 2) could not be assessed in this model. They have been given a consistent value of 0 to allow comparison of the whole profile to other models.

### Exploratory factor analysis (EFA)

An iterative approach to EFA was taken as described in detail in the primary results section. EFA was performed in R, using the “psych” and “stats” packages, with EFA models constructed using combined proliferating and senescent single cell data from each senescence model, as well as with all single target data from all models. Scree plots were generated using factor eigenvalues, and the number of latent factors selected based upon the Kaiser criterion (eigenvalues >1), although this was iteratively assessed. Other parameters included extraction via maximum likelihood and an Oblimin oblique rotation method. Factor loadings were determined through use of a 0.5 loading threshold. Factor scores were calculated using Thompson’s regression method (scores parameter set to regression) and median profiles for each condition displayed as heatmaps.

### Palbociclib treatment of human tumour xenografts

The experimental procedure was approved by the Ethical Committee for Animal Experimentation at the Parc Cientific de Barcelona and by the Government of Catalunya. Nude mice (Hsd:athymic nude-*Foxn1^nu^*) were purchased from Envigo. The animals were kept under a 12–12 h light-dark cycle and allowed unrestricted access to food and water. 10^6^ SK-MEL-103 cells were injected subcutaneously in the dorsolateral flank of 8-week old male athymic nude mice. When tumours became visible at day 8–10 after injection, the mice were randomly assigned to the control or treated experimental groups. Treated mice received 100 mg/kg palbociclib in 50 mM sodium lactate by oral gavage for seven days. Control mice received vehicle only. Once the treatment was complete, the mice were euthanized. The tumours were extracted, fixed with 10% neutral buffered formalin for 24 hours at 4°C and embedded in paraffin for further processing.

### Analysis of palbociclib treatment of human tumour xenografts

Control and palbociclib treated samples were analysed via QuPath open source software [31]. Image type was set as Brightfield (other), with counterstain detected using values 0.651, 0.701 and 0.29 and p21 detected using values 0.269, 0.568 and 0.778. All nuclei were detected via counterstain OD intensity threshold of 0.05 and p21 intensity threshold parameters set at 0.2, 0.4 and 0.6 (1+, 2+ and 3+, respectively) to determine p21 positivity. All features were then extracted apart from those measuring p21 intensity, giving a final list of 27 features (Table 3). Mean standard scores for each feature were then generated using all cells in each treatment condition and presented as heatmaps as described above. Cells identified as p21 positive and negative were then separated and Z-score and standard score profiles generated as described above.

### R analysis

All heatmaps were constructed using the “heatmap.2” package, with distance matrices constructed using Euclidean distances. Hierarchical clustering was then performed via the ward.D2 method. T-distributed stochastic neighbour embedding (t-SNE) dimensionality reduction was performed via the “Rtsne” package with a consistent perplexity parameter of 100 used in each case. T-SNE plots were constructed using the “ggplot2” package. Uniform manifold approximation and projection (UMAP) dimensionality reduction was performed using the step_umap function of the “embed” package. UMAP plots were then constructed using the “ggplot2” package.

### Senescence-associated beta galactosidase assay

Human dermal fibroblasts were fixed with 0.2% glutaraldehyde for 5 min and incubated at pH 6.0 with 1 mg/mL X-gal (10234923, Fisher Scientific, UK), 150 mM NaCl, 2 mM MgCl_2_, 5 mM K_3_Fe(CN)_6_, 5 mM K_4_Fe(CN)_6_ and 40 mM NaPi. This was performed without CO2 for 24 hours at 37°C. Cells were then imaged using a Nikon brightfield tissue culture microscope.

### Statistical analysis

Statistical assessments were performed using Graphpad Prism 7. Experiments using two groups were assessed via unpaired Students *t*-tests and multiple groups via ordinary one-way ANOVA with multiple comparisons. *P* values were represented as: ^*^P < 0.05; ^**^P < 0.01; ^***^P < 0.001; ^****^P < 0.0001. Error bars represent SD of 2 independent experiments unless otherwise stated.

## Supplementary Materials

Supplementary Figures

## References

[1] Hayflick L, Moorhead PS. The serial cultivation of human diploid cell strains. Exp Cell Res. 1961; 25:585–621. 10.1016/0014-4827(61)90192-6 13905658

[2] Serrano M, Hannon GJ, Beach D. A new regulatory motif in cell-cycle control causing specific inhibition of cyclin D/CDK4. Nature. 1993; 366:704–7. 10.1038/366704a0 8259215

[3] d'Adda di Fagagna F, Reaper PM, Clay-Farrace L, Fiegler H, Carr P, Von Zglinicki T, Saretzki G, Carter NP, Jackson SP. A DNA damage checkpoint response in telomere-initiated senescence. Nature. 2003; 426:194–8. 10.1038/nature02118 14608368

[4] Narita M, Nũnez S, Heard E, Narita M, Lin AW, Hearn SA, Spector DL, Hannon GJ, Lowe SW. Rb-mediated heterochromatin formation and silencing of E2F target genes during cellular senescence. Cell. 2003; 113:703–16. 10.1016/s0092-8674(03)00401-x 12809602

[5] Dimri GP, Lee X, Basile G, Acosta M, Scott G, Roskelley C, Medrano EE, Linskens M, Rubelj I, Pereira-Smith O. A biomarker that identifies senescent human cells in culture and in aging skin in vivo. Proc Natl Acad Sci U S A. 1995; 92:9363–7. 10.1073/pnas.92.20.9363 7568133 PMC40985

[6] Coppé JP, Patil CK, Rodier F, Sun Y, Muñoz DP, Goldstein J, Nelson PS, Desprez PY, Campisi J. Senescence-associated secretory phenotypes reveal cell-nonautonomous functions of oncogenic RAS and the p53 tumor suppressor. PLoS Biol. 2008; 6:2853–68. 10.1371/journal.pbio.0060301 19053174 PMC2592359

[7] Wallis R, Mizen H, Bishop CL. The bright and dark side of extracellular vesicles in the senescence-associated secretory phenotype. Mech Ageing Dev. 2020; 189:111263. 10.1016/j.mad.2020.111263 32461143 PMC7347005

[8] Gorgoulis V, Adams PD, Alimonti A, Bennett DC, Bischof O, Bishop C, Campisi J, Collado M, Evangelou K, Ferbeyre G, Gil J, Hara E, Krizhanovsky V, et al. Cellular Senescence: Defining a Path Forward. Cell. 2019; 179:813–27. 10.1016/j.cell.2019.10.005 31675495

[9] Hernandez-Segura A, Nehme J, Demaria M. Hallmarks of Cellular Senescence. Trends Cell Biol. 2018; 28:436–53. 10.1016/j.tcb.2018.02.001 29477613

[10] Caicedo JC, Cooper S, Heigwer F, Warchal S, Qiu P, Molnar C, Vasilevich AS, Barry JD, Bansal HS, Kraus O, Wawer M, Paavolainen L, Herrmann MD, et al. Data-analysis strategies for image-based cell profiling. Nat Methods. 2017; 14:849–63. 10.1038/nmeth.4397 28858338 PMC6871000

[11] Young DW, Bender A, Hoyt J, McWhinnie E, Chirn GW, Tao CY, Tallarico JA, Labow M, Jenkins JL, Mitchison TJ, Feng Y. Integrating high-content screening and ligand-target prediction to identify mechanism of action. Nat Chem Biol. 2008; 4:59–68. 10.1038/nchembio.2007.53 18066055

[12] Ljosa V, Caie PD, Ter Horst R, Sokolnicki KL, Jenkins EL, Daya S, Roberts ME, Jones TR, Singh S, Genovesio A, Clemons PA, Carragher NO, Carpenter AE. Comparison of methods for image-based profiling of cellular morphological responses to small-molecule treatment. J Biomol Screen. 2013; 18:1321–9. 10.1177/1087057113503553 24045582 PMC3884769

[13] Greenberg SB, Grove GL, Cristofalo VJ. Cell size in aging monolayer cultures. In Vitro. 1977; 13:297–300. 10.1007/BF02616174 326658

[14] Cristofalo VJ, Kritchevsky D. Cell size and nucleic acid content in the diploid human cell line WI-38 during aging. Med Exp Int J Exp Med. 1969; 19:313–20. 10.1159/000137216 5408801

[15] Wang E, Gundersen D. Increased organization of cytoskeleton accompanying the aging of human fibroblasts in vitro. Exp Cell Res. 1984; 154:191–202. 10.1016/0014-4827(84)90679-7 6540707

[16] Neurohr GE, Terry RL, Lengefeld J, Bonney M, Brittingham GP, Moretto F, Miettinen TP, Vaites LP, Soares LM, Paulo JA, Harper JW, Buratowski S, Manalis S, et al. Excessive Cell Growth Causes Cytoplasm Dilution And Contributes to Senescence. Cell. 2019; 176:1083–97.e18. 10.1016/j.cell.2019.01.018 30739799 PMC6386581

[17] Sadaie M, Dillon C, Narita M, Young AR, Cairney CJ, Godwin LS, Torrance CJ, Bennett DC, Keith WN, Narita M. Cell-based screen for altered nuclear phenotypes reveals senescence progression in polyploid cells after Aurora kinase B inhibition. Mol Biol Cell. 2015; 26:2971–85. 10.1091/mbc.E15-01-0003 26133385 PMC4551313

[18] Zhao H, Halicka HD, Traganos F, Jorgensen E, Darzynkiewicz Z. New biomarkers probing depth of cell senescence assessed by laser scanning cytometry. Cytometry A. 2010; 77:999–1007. 10.1002/cyto.a.20983 20939035 PMC2977923

[19] González-Gualda E, Baker AG, Fruk L, Muñoz-Espín D. A guide to assessing cellular senescence in vitro and in vivo. FEBS J. 2021; 288:56–80. 10.1111/febs.15570 32961620

[20] Wallis R, Josipovic N, Mizen H, Robles-Tenorio A, Tyler EJ, Papantonis A, Bishop CL. Isolation methodology is essential to the evaluation of the extracellular vesicle component of the senescence-associated secretory phenotype. J Extracell Vesicles. 2021; 10:e12041. 10.1002/jev2.12041 33659050 PMC7892802

[21] Tyler EJ, Gutierrez Del Arroyo A, Hughes BK, Wallis R, Garbe JC, Stampfer MR, Koh J, Lowe R, Philpott MP, Bishop CL. Early growth response 2 (EGR2) is a novel regulator of the senescence programme. Aging Cell. 2021; 20:e13318. 10.1111/acel.13318 33547862 PMC7963333

[22] Borgdorff V, Lleonart ME, Bishop CL, Fessart D, Bergin AH, Overhoff MG, Beach DH. Multiple microRNAs rescue from Ras-induced senescence by inhibiting p21(Waf1/Cip1). Oncogene. 2010; 29:2262–71. 10.1038/onc.2009.497 20101223

[23] Brideau C, Gunter B, Pikounis B, Liaw A. Improved statistical methods for hit selection in high-throughput screening. J Biomol Screen. 2003; 8:634–47. 10.1177/1087057103258285 14711389

[24] Snook SC, Gorsuch RL. Component Analysis Versus Common Factor Analysis: A Monte Carlo Study. Psychological Bulletin. 1989; 106:148–54. 10.1037/0033-2909.106.1.148

[25] Kim HJ. Common factor analysis versus principal component analysis: choice for symptom cluster research. Asian Nurs Res (Korean Soc Nurs Sci). 2008; 2:17–24. 10.1016/S1976-1317(08)60025-0 25031108

[26] Kaufman JD, Dunlap WP. Determining the number of factors to retain: a Windows-based FORTRAN-IMSL program for parallel analysis. Behav Res Methods Instrum Comput. 2000; 32:389–95. 10.3758/bf03200806 11029810

[27] Kaiser HF. The Application of Electronic Computers to Factor Analysis. Educational and Psychological Measurement. 1960; 20:141–51. 10.1177/001316446002000116

[28] Teo YV, Rattanavirotkul N, Olova N, Salzano A, Quintanilla A, Tarrats N, Kiourtis C, Müller M, Green AR, Adams PD, Acosta JC, Bird TG, Kirschner K, et al. Notch Signaling Mediates Secondary Senescence. Cell Rep. 2019; 27:997–1007.e5. 10.1016/j.celrep.2019.03.104 31018144 PMC6486482

[29] Hoare M, Ito Y, Kang TW, Weekes MP, Matheson NJ, Patten DA, Shetty S, Parry AJ, Menon S, Salama R, Antrobus R, Tomimatsu K, Howat W, et al. NOTCH1 mediates a switch between two distinct secretomes during senescence. Nat Cell Biol. 2016; 18:979–92. 10.1038/ncb3397 27525720 PMC5008465

[30] Passos JF, Nelson G, Wang C, Richter T, Simillion C, Proctor CJ, Miwa S, Olijslagers S, Hallinan J, Wipat A, Saretzki G, Rudolph KL, Kirkwood TB, von Zglinicki T. Feedback between p21 and reactive oxygen production is necessary for cell senescence. Mol Syst Biol. 2010; 6:347. 10.1038/msb.2010.5 20160708 PMC2835567

[31] Bankhead P, Loughrey MB, Fernández JA, Dombrowski Y, McArt DG, Dunne PD, McQuaid S, Gray RT, Murray LJ, Coleman HG, James JA, Salto-Tellez M, Hamilton PW. QuPath: Open source software for digital pathology image analysis. Sci Rep. 2017; 7:16878. 10.1038/s41598-017-17204-5 29203879 PMC5715110

[32] Kohli J, Wang B, Brandenburg SM, Basisty N, Evangelou K, Varela-Eirin M, Campisi J, Schilling B, Gorgoulis V, Demaria M. Algorithmic assessment of cellular senescence in experimental and clinical specimens. Nat Protoc. 2021; 16:2471–98. 10.1038/s41596-021-00505-5 33911261 PMC8710232

[33] Hwang ES, Yoon G, Kang HT. A comparative analysis of the cell biology of senescence and aging. Cell Mol Life Sci. 2009; 66:2503–24. 10.1007/s00018-009-0034-2 19421842 PMC11115533

[34] Kusumoto D, Seki T, Sawada H, Kunitomi A, Katsuki T, Kimura M, Ito S, Komuro J, Hashimoto H, Fukuda K, Yuasa S. Anti-senescent drug screening by deep learning-based morphology senescence scoring. Nat Commun. 2021; 12:257. 10.1038/s41467-020-20213-0 33431893 PMC7801636

[35] Chandrasekaran SN, Ceulemans H, Boyd JD, Carpenter AE. Image-based profiling for drug discovery: due for a machine-learning upgrade? Nat Rev Drug Discov. 2021; 20:145–59. 10.1038/s41573-020-00117-w 33353986 PMC7754181

[36] Bray MA, Singh S, Han H, Davis CT, Borgeson B, Hartland C, Kost-Alimova M, Gustafsdottir SM, Gibson CC, Carpenter AE. Cell Painting, a high-content image-based assay for morphological profiling using multiplexed fluorescent dyes. Nat Protoc. 2016; 11:1757–74. 10.1038/nprot.2016.105 27560178 PMC5223290

[37] Hari P, Millar FR, Tarrats N, Birch J, Quintanilla A, Rink CJ, Fernández-Duran I, Muir M, Finch AJ, Brunton VG, Passos JF, Morton JP, Boulter L, Acosta JC. The innate immune sensor Toll-like receptor 2 controls the senescence-associated secretory phenotype. Sci Adv. 2019; 5:eaaw0254. 10.1126/sciadv.aaw0254 31183403 PMC6551188

