# Supplementary material for: Senescence-associated morphological profiles (SAMPs): an image-based phenotypic profiling method for evaluating the inter and intra model heterogeneity of senescence
Source: Aging (Albany NY). 2022 May 16;14(10):4220–46. doi: 10.18632/aging.204072 (PMC9186762; doi:10.18632/aging.204072)
Supplement: Supplementary Figures [file aging-14-204072-s001.pdf]

SUPPLEMENTARY FIGURES

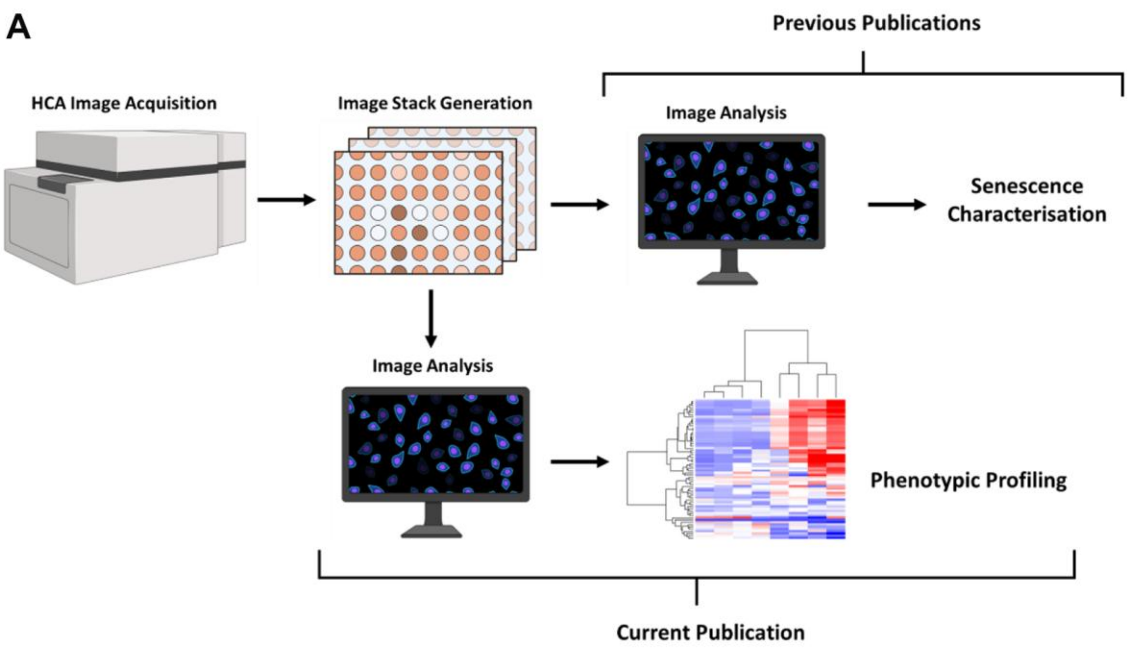

B

Model	Cell Type	Publication	Senescence Markers								
			Proliferation Arrest	Nuclear Morphology	Cellular Morphology	p21 Expression	p16 Expression	SASP	SAHFs	8-Oxoguanine	Lamin B1
OIS Induction	IMR90	(Wallis *et al.*, 2021)	✓	✓	✓	✓	✓	✓	✓		
Paracrine Senescence	IMR90	(Wallis *et al.*, 2021)	✓	✓	✓						
Replicative Senescence	HMFs	(Tyler *et al.*, 2021)	✓	✓	✓	✓	✓	✓		✓	✓
OIS Induction	HMEC	(Borgdorff *et al.*, 2010)	✓	✓	✓	✓					

Supplementary Figure 1. Workflow for use of established image stacks. (**A**) Image stacks were previously generated through high content microscopy for oncogene-indcued senescence (IMR90) , paracrine senescence (IMR90), replicative senescence (HMFs) and oncogene-indcued senescence (HMEC) models. These were then used to characterise a range of conventional senecence hallmarks. For this work, these image stacks were re-mined utilising phenotypic profiling for morphological assessment. Figure created with BioRender. (**B**) Summary of senescence markers used in previous publications for confirmatory senescence identification.

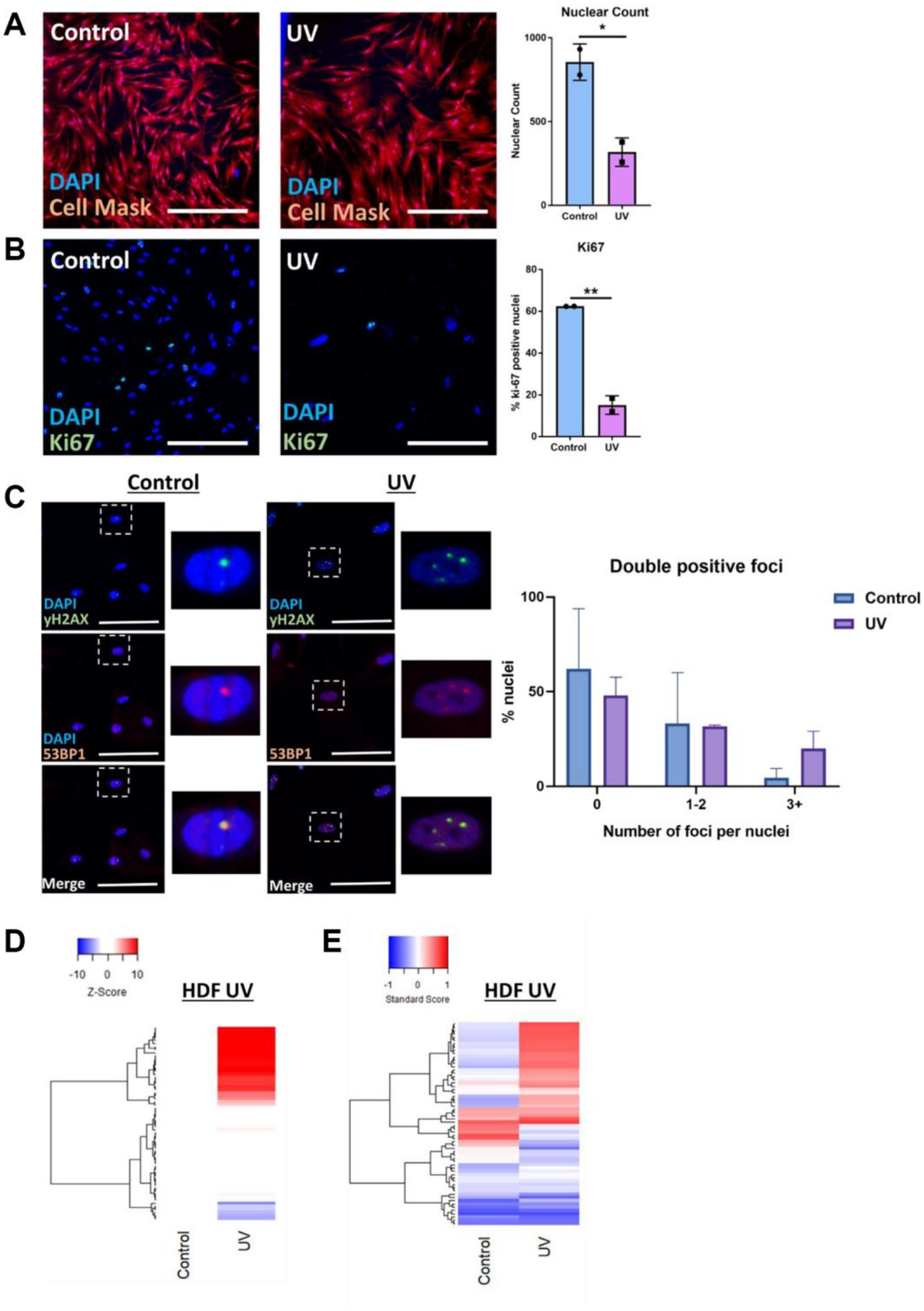

Supplementary Figure 2. Characterisation of UV-induced senescence in human dermal fibroblasts. (**A**) Immunofluorescence staining and quantitation of DAPI (blue) and Cell Mask (Red) in UV-induced senescence model. *N* = 2. Scale bar = 100 μm. (**B**) Immunofluorescence staining and quantitation of DAPI (blue) and Ki67 (green) in UV-induced senescence model. *N* = 2. Scale bar = 100 μm; (**C**) Immunofluorescence staining of DAPI (blue), γH2AX foci (green) and 53BP1 (red) in UV-induced senescence model. *N* = 2. Scale bar = 50 μm. (**D**) Z-score profile heatmap of UV-induced senescence model. Y-axis comprises 62 morphological features (Red = positive modulation, Blue = negative modulation), White = no change) (**E**) Standard score profile heatmap of UV-induced senescence model. Y-axis comprises 62 morphological features (Red = positive modulation, Blue = negative modulation), White = no change). Proliferating condition (HDFs treated with 0 mJ/cm^2 UVB; Control). Senescent condition (HDFs treated with 6mJ/cm^2 UVB; UV).

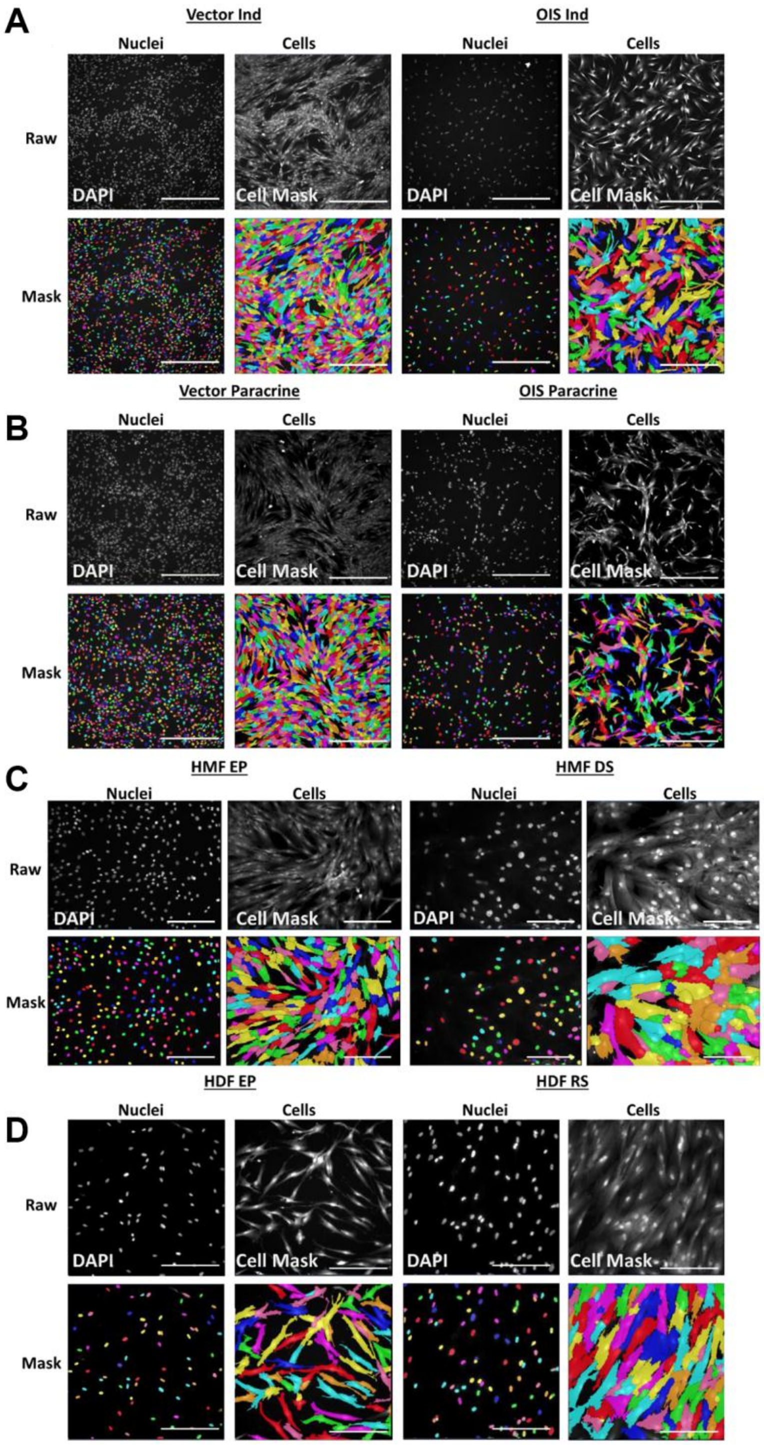

Supplementary Figure 3. InCarta mask generation. Representative raw images from each senescence model alongside nuclear and cellular masks generated via InCarta high content analysis (HCA) software. (**A**) Oncogene-induced senescence model. Proliferating condition: vector induction (Vector Ind), Senescence condition: OIS induction (OIS Ind). Scale bars = 500 µm (**B**) Paracrine senescence model. Proliferating condition: Vector paracrine, Senescence condition: OIS Paracrine. Scale bars = 500 µm (**C**) Human mammary fibroblast (HMF) replicative senescence model. Proliferating condition: Early proliferating HMFs (HMF EP), Senescence condition: Deep senescence HMFs (HMF DS). Scale bars = 250 µm (**D**) Human dermal fibroblast (HDF) replicative senescence model. Proliferating condition: Early proliferating HDFs (HDF EP), Senescence condition: Deep senescence HDFs (HDF DS). Scale bars = 250 µm.

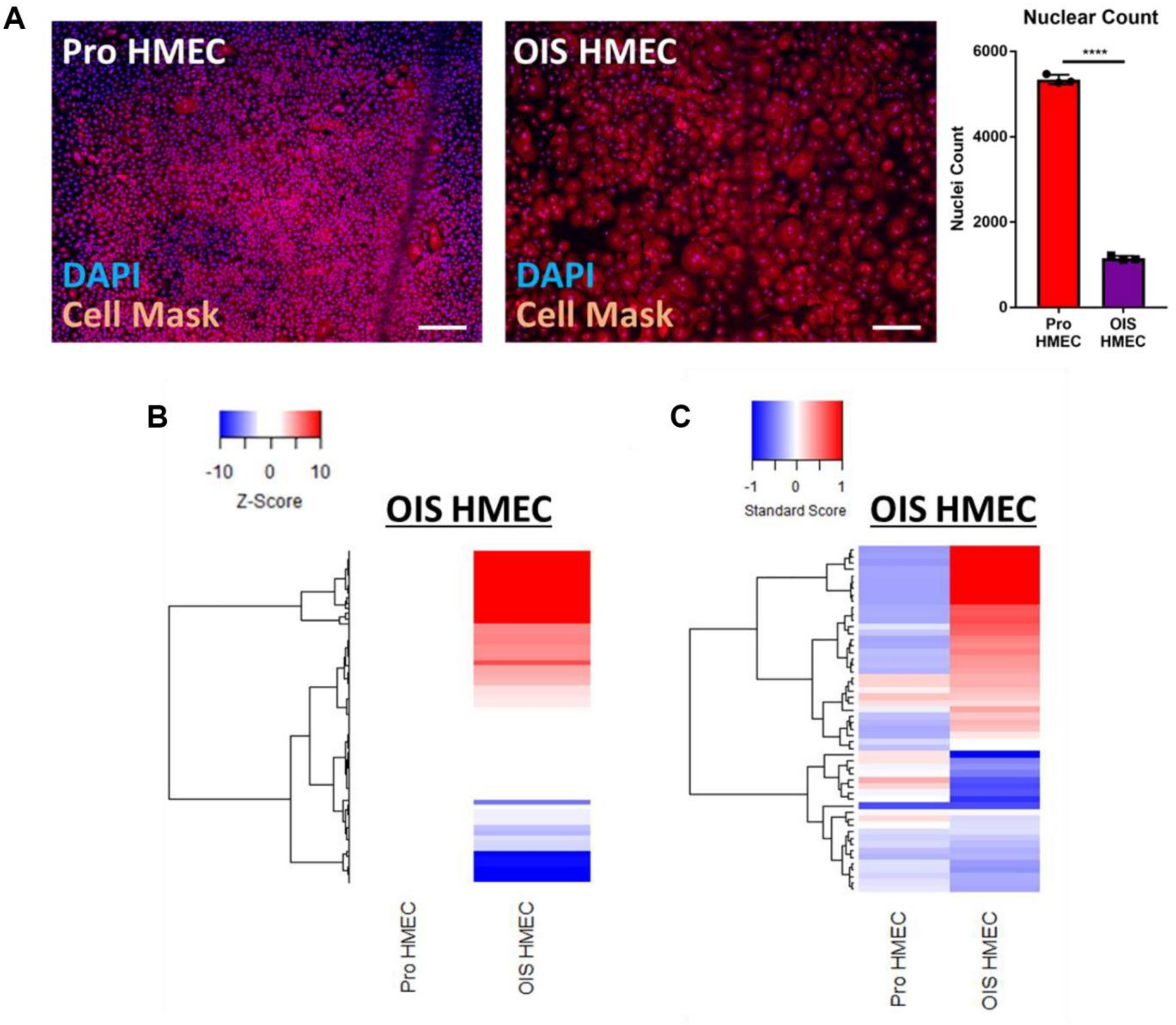

Supplementary Figure 4. Characterisation of OIS in human mammary epithelial cells (HMEC). (**A**) Immunofluorescence staining and quantitation of DAPI (blue) and Cell Mask (Red) inhuman mammary epithelial cell (HMEC) oncogene-induced senescence (OIS) model. *N* = 3. Scale bar = 500 μm. (**B**) Z-score profile heatmap of HMEC OIS model. Y-axis comprises 62 morphological features (Red = positive modulation, Blue = negative modulation), White = no change) (**C**) Standard score profile heatmap HMEC OIS model. Y-axis comprises 62 morphological features (Red = positive modulation, Blue = negative modulation), White = no change). Proliferating condition (ER:RAS HMEC without tamoxifen induction; Pro HMEC). Senescent condition (ER:RAS HMEC with tamoxifen induction; OIS HMEC).

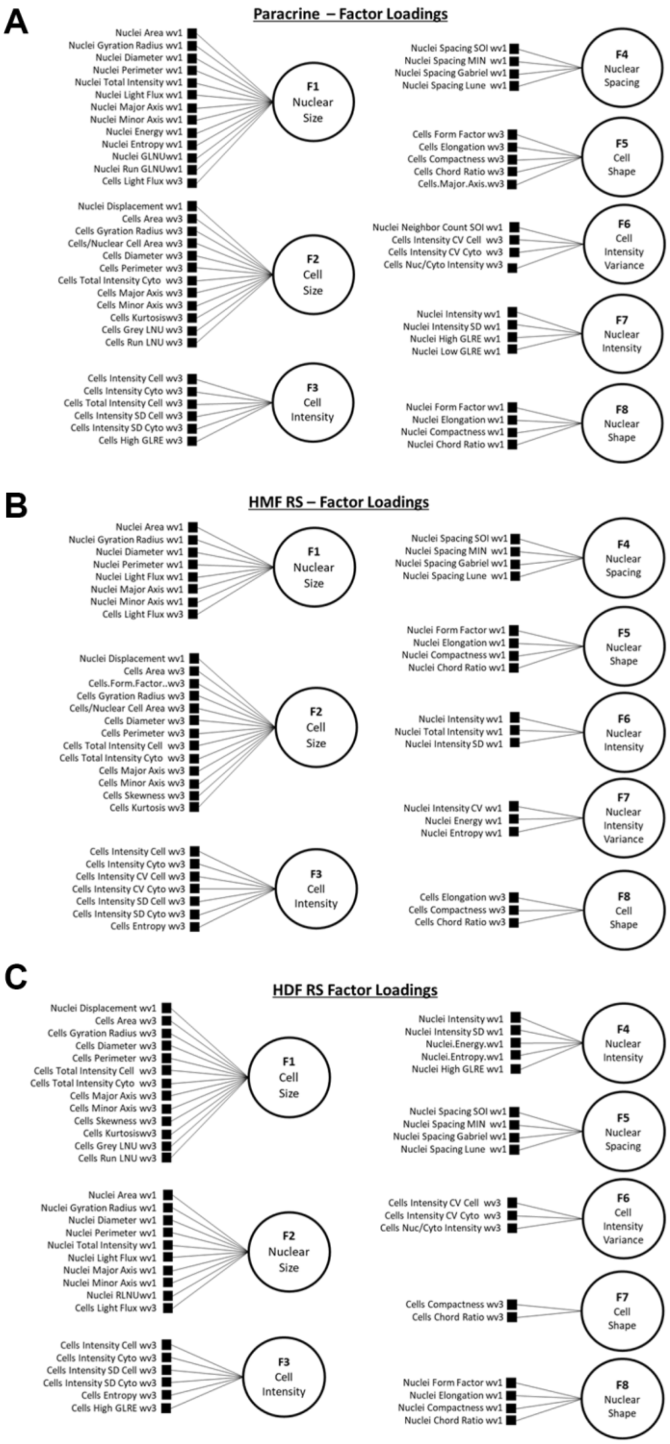

Supplementary Figure 5. Exploratory factor analysis (EFA) factor loading diagrams. (**A**–**C**) Factor loading diagrams for EFA models constructed using data from paracrine senescence, HMF replicative senescence and HDF replicative senescence models. Factor designations are also indicated.

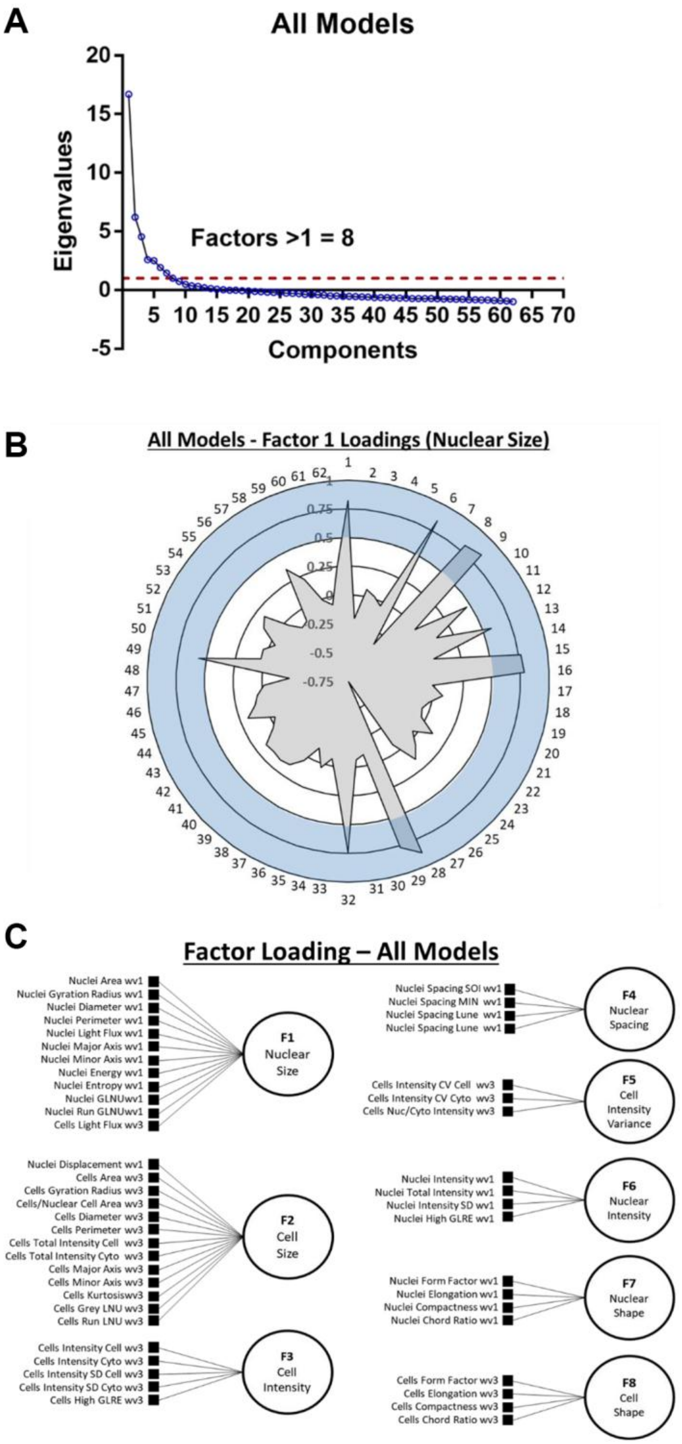

Supplementary Figure 6. EFA model using single target data from all senescence models. (**A**) Scree plot for combined single target data from all senescence models. Red line indicates eigenvalue = 1. (**B**) Polar plot of factor loading values for factor 1 from all senescence models EFA model (designated Nuclear Size). 1–62 refer to features (Table 2). Blue shaded area indicates factor loading threshold of 0.5. (**C**) Factor loading diagram for all senescence models EFA model with factor designations.

A

Latent Factor	Extracted Feature	Literature Precedent
Nuclear Size	Nuclear Area (1)	(Zhao *et al.*, 2010; Studencka and Schaber, 2017)
Nuclear Shape	Nuclear Form Factor (2)	(Matsumura, Zerrudo and Hayflick, 1979; Sadaie *et al.*, 2015)
Nuclear Intensity	Nuclear Intensity (10)	(Zhao and Darzynkiewicz, 2013; Roukos *et al.*, 2015)
Nuclear Intensity (Variance)	Nuclear Intensity CoV (12)	(Narita *et al.*, 2003; Di Micco *et al.*, 2011)
Nuclear Spacing	Nuclear Spacing SOI (17)	(Hayflick and Moorhead, 1961)
Cell Size	Cell Area (33)	(Cristofalo and Kritchevsky, 1969; Biran *et al.*, 2017)
Cell Shape	Cell Form Factor (34)	(Druelle *et al.*, 2016; Cormenier *et al.*, 2018)
Cell Intensity	Cell Intensity Cell (42)	(Neurohr *et al.*, 2019)
Cell Intensity (Variance)	Cell Intensity CoV Cell (46)	-

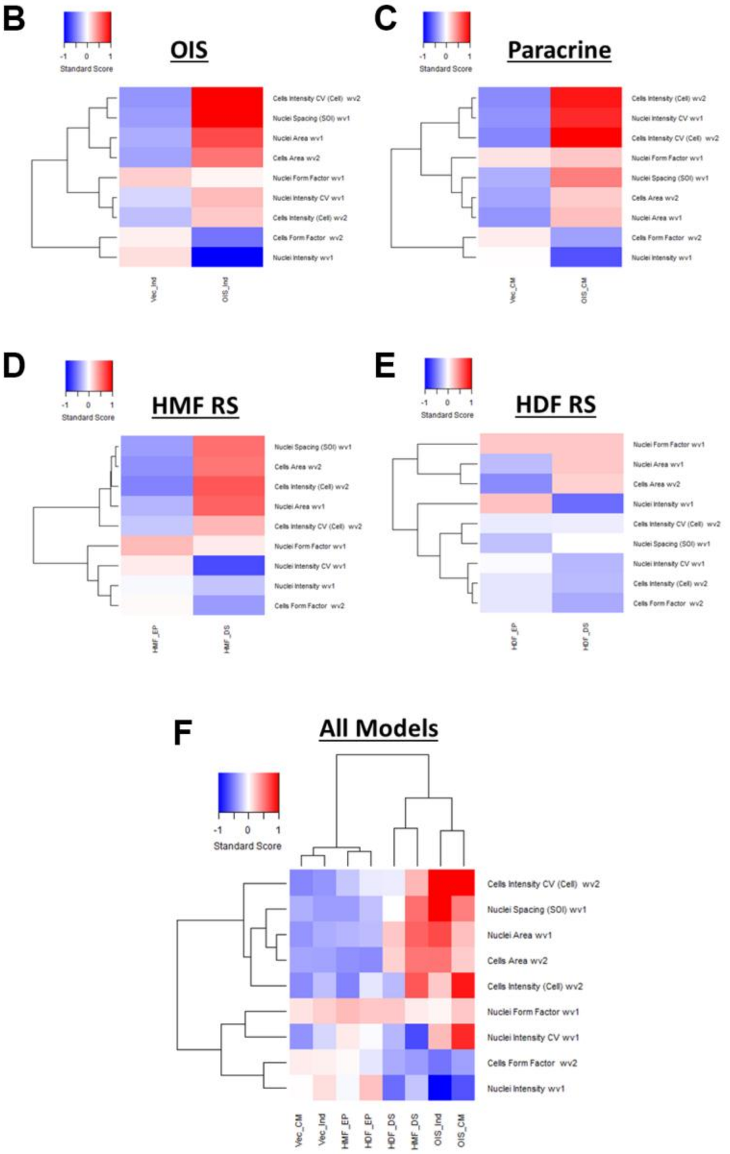

Supplementary Figure 7. Representative profiles. (**A**) Table indicating senescence-associated latent factors and selected representative features. Literature precedent for use of feature in senescence characterisation included. (**B**–**E**) Standard score profile heatmaps for oncogene-induced senescence (OIS), paracrine senescence (Paracrine), HMF replicative senescence (HMF RS) and HDF replicative senescence (HDF RS) models. Y-axis comprises standard-scores for the 8 extracted features above (Red = positive modulation, Blue = negative modulation), White = no change). Proliferating conditions: vector induction (Vec_Ind), vector conditioned media (Vec_CM), HMF early proliferating (HMF_EP) and HDF early proliferating (HDF_EP). Senescence conditions: OIS induction (OIS_Ind), OIS conditioned media (OIS_CM), HMF deep senescence (HMF_DS), HDF deep senescence (HDF_DS). (**F**) Summary standard-score profile heat map and hierarchical clustering of all proliferating and senescence conditions. Y-axis comprises standard-scores for the 8 extracted features above.
